# Physiological acclimatization in Hawaiian corals following a 22-month shift in baseline seawater temperature and pH

**DOI:** 10.1038/s41598-022-06896-z

**Published:** 2022-03-10

**Authors:** Rowan H. McLachlan, James T. Price, Agustí Muñoz-Garcia, Noah L. Weisleder, Stephen J. Levas, Christopher P. Jury, Robert J. Toonen, Andréa G. Grottoli

**Affiliations:** 1 School of Earth Sciences, The Ohio State University, 125 South Oval Mall, Columbus, OH 43210 USA; 2 Department of Microbiology, Oregon State University, 2820 SW Campus Way, Corvallis, OR 97331 USA; 3 Department of Evolution, Ecology and Organismal Biology, The Ohio State University at Mansfield, 1760 University Dr., Mansfield, OH 44906 USA; 4 Department of Physiology and Cell Biology, The Ohio State University, 473 West 12th Avenue, Columbus, OH 43210 USA; 5 Geography, Geology and Environmental Science, University of Wisconsin – Whitewater, 800 W. Main Street, Whitewater, WI 53190 USA; 6 Hawaiʻi Institute of Marine Biology, University of Hawaiʻi at Mānoa, 46-007 Lilipuna Road, Kāneʻohe, HI 96744 USA

**Keywords:** Climate-change ecology, Ecophysiology, Marine biology, Climate-change impacts

## Abstract

Climate change poses a major threat to coral reefs. We conducted an outdoor 22-month experiment to investigate if coral could not just survive, but also physiologically cope, with chronic ocean warming and acidification conditions expected later this century under the Paris Climate Agreement. We recorded survivorship and measured eleven phenotypic traits to evaluate the holobiont responses of Hawaiian coral: color, Symbiodiniaceae density, calcification, photosynthesis, respiration, total organic carbon flux, carbon budget, biomass, lipids, protein, and maximum *Artemia* capture rate. Survivorship was lowest in *Montipora capitata* and only some survivors were able to meet metabolic demand and physiologically cope with future ocean conditions. Most *M. capitata* survivors bleached through loss of chlorophyll pigments and simultaneously experienced increased respiration rates and negative carbon budgets due to a 236% increase in total organic carbon losses under combined future ocean conditions. *Porites compressa* and *Porites lobata* had the highest survivorship and coped well under future ocean conditions with positive calcification and increased biomass, maintenance of lipids, and the capacity to exceed their metabolic demand through photosynthesis and heterotrophy. Thus, our findings show that significant biological diversity within resilient corals like *Porites*, and some genotypes of sensitive species, will persist this century provided atmospheric carbon dioxide levels are controlled. Since *Porites* corals are ubiquitous throughout the world’s oceans and often major reef builders, the persistence of this resilient genus provides hope for future reef ecosystem function globally.

## Introduction

Coral reefs are threatened worldwide due to the environmental impacts of climate change^1^. Rising seawater temperature due to global warming is considered to be the greatest threat to corals as it induces mass coral bleaching within and across tropical reef regions^2–4^. Thermal stress during ocean warming events causes a breakdown of the coral-algal symbiosis, and as a result, the algal endosymbionts (family Symbiodiniaceae) are expelled leaving corals white in color and unable to obtain fixed carbon through photosynthesis^5,6^. If thermal stress is prolonged, corals are unable to recover their Symbiodiniaceae partners which can lead to decreases in coral health, increases in disease prevalence, and high levels of coral mortality^7,8^. The second global threat to coral reefs is ocean acidification. Reduced seawater pH can cause dissolution and weakening of coral skeletons, has been shown to slow or even stop calcification, and can also exacerbate the negative effects of temperature stress on some species (e.g.,^9–12^). Overall, it is predicted that seawater temperature will increase by another 0.2–3.5 °C with concomitant drops in pH of 0.1–0.3 units by the year 2100, depending on the CO_2_ emissions scenario^13^.

While the response of corals to future ocean stress of elevated temperature coupled with reduced pH has been the focus of a growing number of studies over the last decade (e.g.,^12,14,15^), the physiological traits that allow the coral holobiont (i.e., animal host, algal endosymbionts, and associated microbiome) to cope with multi-annual exposure to baseline shifts in both stressors, coupled with repeat summer heat-wave events as is expected later this century, is unknown. Here, we define holobiont “coping” as survival under the dual stress of ocean warming and acidification conditions, coupled with the maintenance of coral pigmentation, net positive calcification, maintenance of tissue biomass and energy reserves, and sufficient fixed carbon acquisition through photosynthesis and heterotrophy to meet metabolic demand. These traits were selected as an increase in coral whiteness is a visible sign of bleaching due to loss of chlorophyll and Symbiodiniaceae cells^16,17^. Patterns in calcification rates and tissue biomass are indicative of energy allocation between skeletal structures and somatic tissue^18^. Energy reserves are known to be important indicators of coral health^19–22^ which corals rely upon during times of metabolic energy deficits^23,24^. Heterotrophy is critical to coral lipid synthesis and tissue rebuilding following bleaching^25,26^. Finally, the inability of corals to meet metabolic demand is a clear indicator of stress^27–29^. Here we address two overarching questions: (1) *Which corals will survive chronic future ocean conditions?* and (2) *How well do survivors cope with future ocean conditions?* We investigated three of the most abundant coral species in Hawaiʻi (*Montipora capitata*, *Porites compressa*, and *Porites lobata*). These species experience differential resilience to thermal stress and/or ocean acidification (e.g.,^14,30,31^) and varying capacities to recover from bleaching (e.g.,^26,28^), making them ideal candidates to evaluate the likely responses of Hawaiian corals to future ocean conditions.

## Methods

### Coral species, sample collection, and acclimation

**Figure 1:**
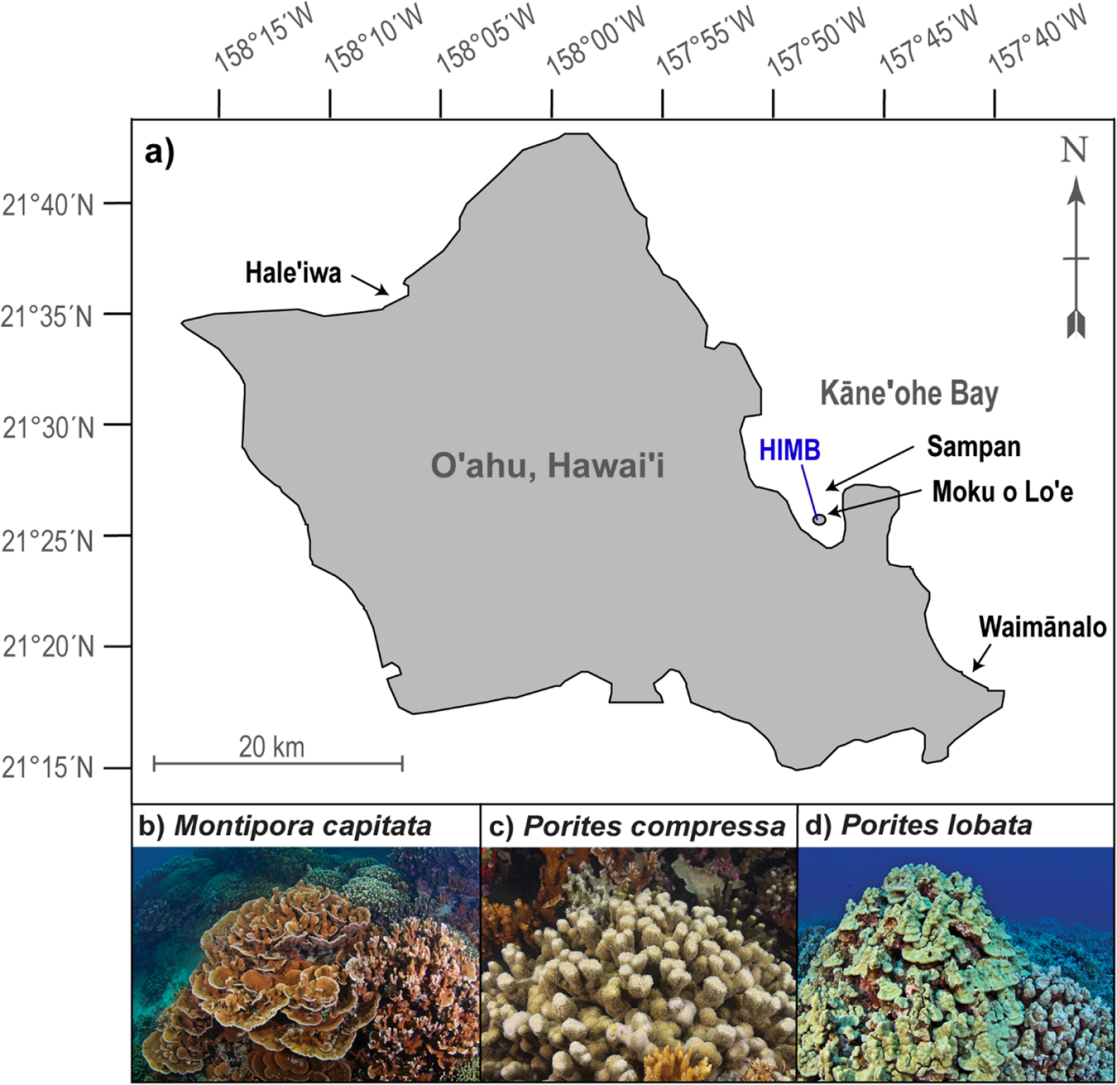
(**a**) Map of four coral collection sites around the island of Oʻahu, Hawaiʻi (black text), experimental location at the Hawaiʻi Institute of Marine Biology (HIMB, blue text), and photos of the study species (**b**) *M. capitata*, (**c**) *P. compressa*, and (**d**) *P. lobata.* Photographs courtesy of Keoki Stender.

The corals *Montipora capitata* (branching and encrusting), *Porites compressa* (branching), and *Porites lobata* (massive) were collected at 2 ± 1 m depth between 29 August and 11 November 2015 from four reef sites around the island of Oʻahu, Hawaiʻi: Moku o Loʻe and Sampan within Kāneʻohe Bay, Waimānalo, and Haleʻiwa (Fig. 1). *P. lobata* was not found at Moku o Loʻe and was not collected there. This broad spatial sampling of corals helped to ensure that a representative sample of the genetic variation present in these species from Oʻahu was included in the study. Six genets of each species were collected at each site using a hammer and chisel for a total of 66 genets (24 parent colonies for *M. capitata*, 24 parent colonies for *P. compressa*, and 18 parent colonies for *P. lobata*) (Table S1). Species-specific microsatellite markers (developed by^32,33^) were used to genotype all corals and ensure that they were genetically distinct. After removal from the reef, genets were placed in individual plastic bags filled with seawater from the collection site, and transported back to the Hawaiʻi Institute of Marine Biology (HIMB, Fig. 1a). Four clonal ramets were cut from each genet using a band saw, and each ramet was mounted on a labelled ceramic plug using cyanoacrylate gel. The 264 ramets (i.e., 66 genets × 4 ramets, Table S1) were distributed among the experimental outdoor flow-through mesocosm tanks, and allowed to recover and acclimate to the mesocosm system under ambient reef-supplied flow-through seawater for at least 12 weeks until 31 January 2016. Shade cloth above the mesocosm tanks attenuated sunlight by 30% to provide irradiance like that at collection depth, with a maximum instantaneous irradiance of ~ 1730 µmol m^−2^ s^−1^^34^. A complete timeline of experimental procedures can be found in Fig. S1.

### Mesocosm experiment

**Figure 2:**
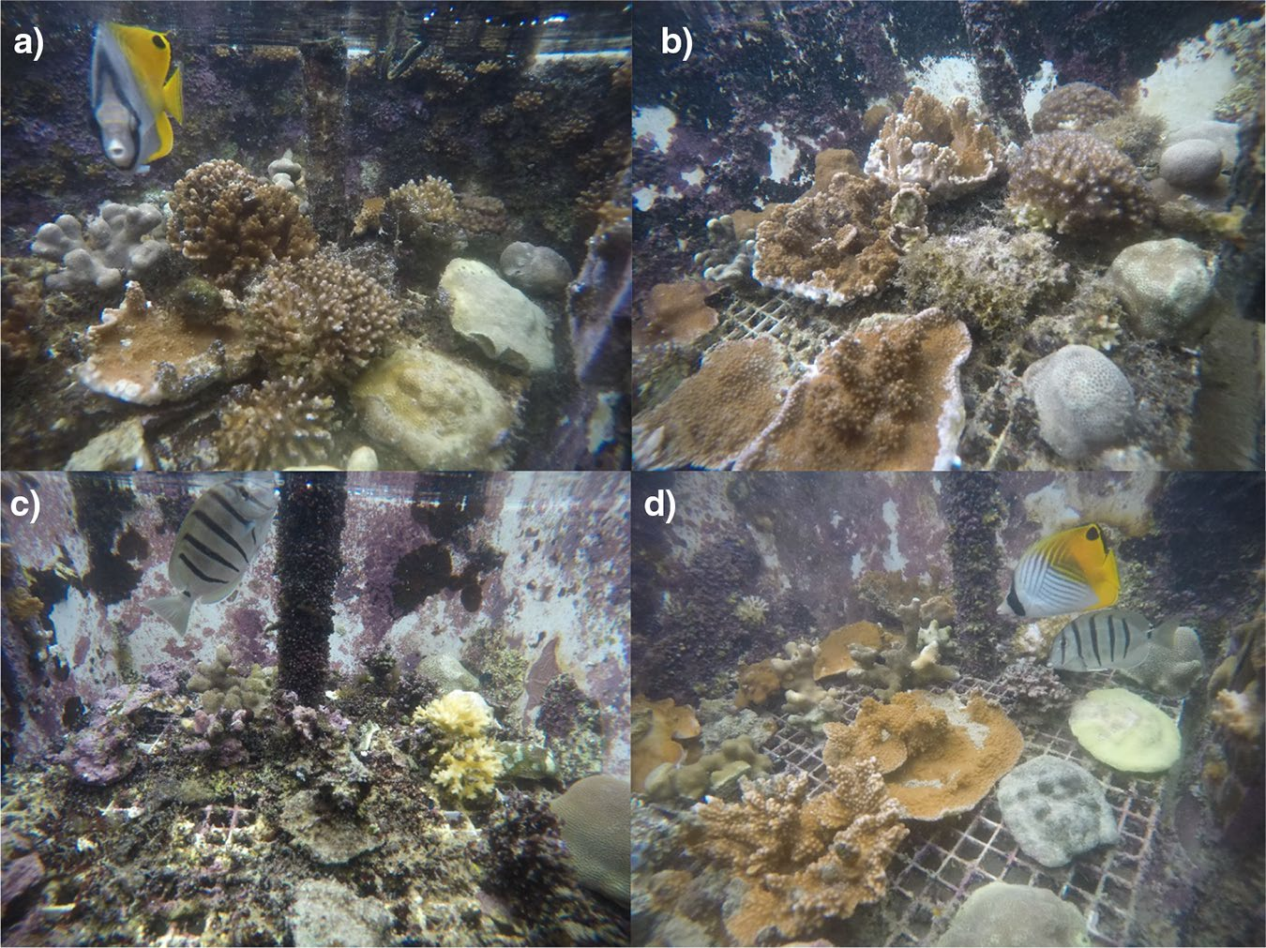
Representative photos of the mesocosms after twenty-two months of exposure to: (**a**) control, (**b**) ocean acidification, (**c**) ocean warming, and (**d**) future ocean treatment conditions.

**Figure 3:**
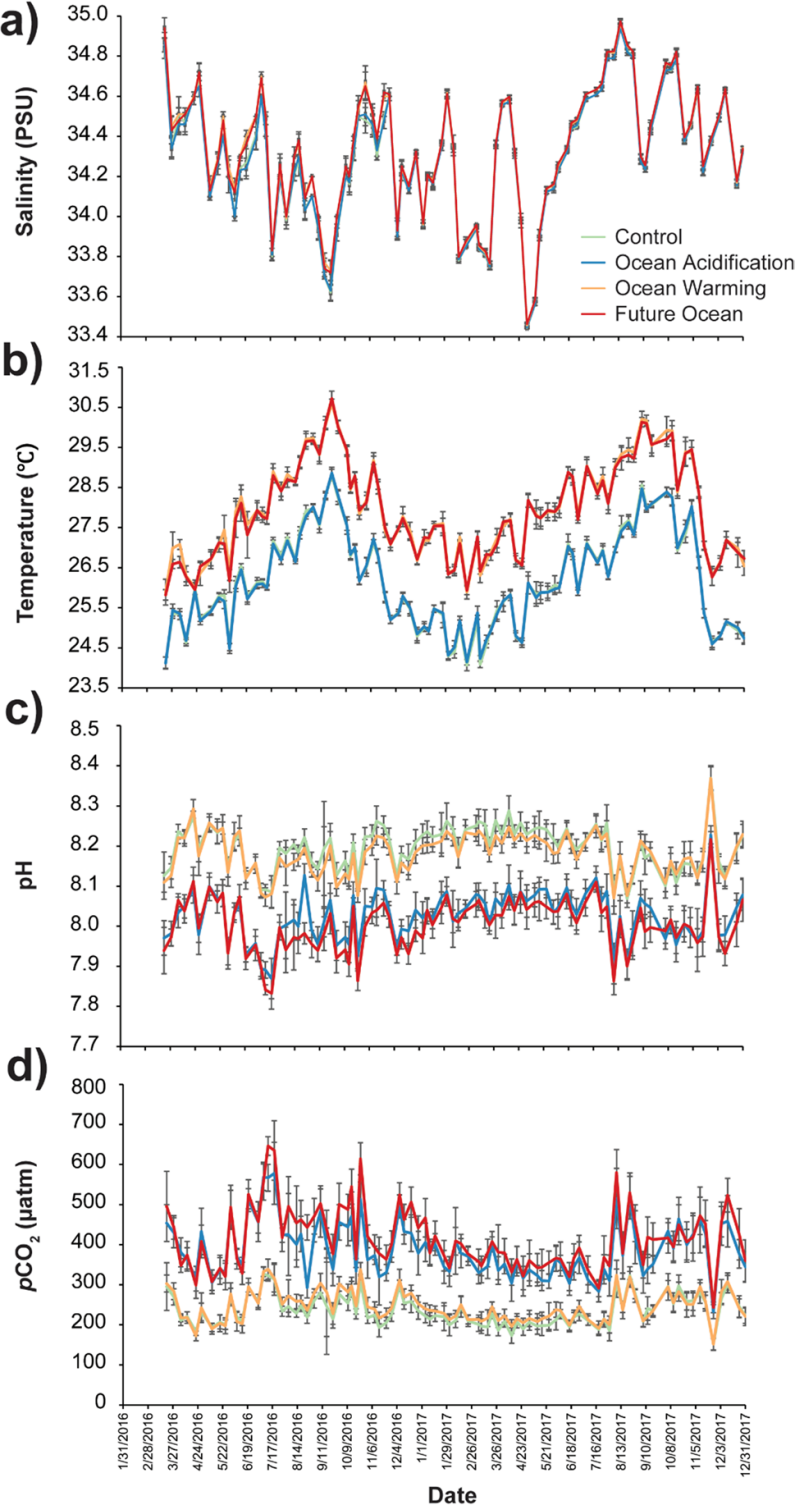
Weekly mean ± 1 SD mid-day (12:00) (**a**) salinity, (**b**) temperature, (**c**) pH, and (**d**) *p*CO_2_ in each experimental treatment: control (green), ocean acidification (blue), ocean warming (orange) and combined future ocean (red) throughout the 22-month experimental period. Dates provided as MM/DD/YYYY.

This study was part of a larger mesocosm experiment that is comprehensively described previously^35^. Meta-data regarding the experimental methods can be found in Table S2 and a detailed description of the experimental methods are provided in the Supplemental Text. Briefly, the experiment consisted of four treatments (n = 10 mesocosms per treatment) as follows: control (present-day temperature with present-day *p*CO_2_), ocean acidification (present-day temperature with + 350 μatm *p*CO_2_), ocean warming (+ 2 ℃ with present-day *p*CO_2_), and combined future ocean conditions (+ 2 °C with + 350 μatm *p*CO_2_). The elevated temperature and *p*CO_2_ levels are consistent with current commitments under the Paris Climate Agreement^36^. The ramets of *M. capitata*, *P. compressa*, and *P. lobata* were distributed among the 40 outdoor flow-through mesocosm tanks (70 L, 50 × 50 × 30 cm) at HIMB such that one ramet per genet was present within each of the four treatment conditions. Corals were maintained under experimental conditions for 22 months from 20 February 2016 to 13 December 2017 for a total of 662 days (Fig. S1). Representative photos of the mesocosm tanks for each treatment at the end of the experimental period are shown in Fig. 2. Salinity, temperature, *p*CO_2_, and pH were measured at mid-day in each mesocosm once weekly and treatment average weekly values (± 1SD) were plotted (Fig. 3). This is a long-term experiment as defined by McLachlan et al.^37^ and Grottoli et al.^38^ and the longest dual stress (i.e., combined ocean warming and acidification) experiment on corals to date (Table S3).

Coral fragments were photographed for surface area and ramet whiteness analysis, and buoyant weighed for growth rate on the weeks of 20 March 2016 and 27 November 2017 corresponding to one month after the target temperature and pH conditions were reached and the end of the experimental period, respectively (Fig. S1). Observed growth rates replicated near maximal growth rates observed in these species on the reef^39^, indicating that the mesocosms closely replicated reef conditions needed for optimal growth. Thus, findings in this study are likely to reflect responses expected under reef conditions. During the last 20 days of the experimental period (23 November–13 December 2017) the following live physiological measurements were conducted on all surviving coral ramets: photosynthesis, respiration, total organic carbon (TOC) flux, and maximum *Artemia* feeding capacity (Fig. S1). Equations associated with live measurements can be found in Table S4. Then, all surviving coral fragments were sacrificed by freezing at − 20 ℃. Samples were transported on dry ice to the Ohio State University (OH, USA) where they were stored at − 80 ℃ awaiting further analyses of biomass, lipids, proteins, Symbiodiniaceae density, and surface area according to methods published in protocols.io^40–44^. At least 10% of all live and post-sacrifice physiological measurements were made in duplicate. Additional details of the laboratory analysis methods are provided in the Supplemental Text.

In addition, the carbon budget of each coral ramet was calculated to determine the proportionate contribution of photosynthesis and heterotrophy to total metabolic demand (i.e., respiration). Photosynthesis and total respiration rates were used to calculate the percent Contribution of Zooxanthellae (i.e., Symbiodiniaceae) to Animal Respiration (CZAR)^45^, while total respiration and nighttime TOC flux rates were used to calculate the percent Contribution of Heterotrophy from TOC to Animal Respiration (CHAR_TOC_)^46^. *Artemia* feeding capacity was not used to calculate CHAR_zoop_ as the *Artemia* concentrations were not representative of reef zooplankton densities or mesocosm zooplankton densities. The Contribution of the Total acquired fixed carbon relative to Animal Respiration (CTAR)^27^ was calculated as the sum of CZAR and CHAR_TOC_. However, we acknowledge that this is likely an underestimate of CTAR as it does not account for heterotrophic carbon derived from zooplankton nor any potential gains or losses in CHAR_TOC_ that may have occurred during the day. Equations associated with carbon budget parameters can be found in Table S4.

### Data analysis

To test for the effects of species temperature, and *p*CO_2_ on survivorship, survivorship data were analyzed using a generalized linear model with a binomial error distribution. To identify the physiological mechanisms underlying the ability of survivors of each species to cope (or not cope) with future ocean conditions, multivariate statistical analyses were performed. Ten physiological traits were standardized prior to the construction of Euclidean distance resemblance matrices. Data were visualized using non-parametric multidimensional scaling (NMDS) plots for each species. The effects of temperature and *p*CO_2_ (two-way analysis), and treatment (one-way analysis) on coral multivariate physiological profiles were investigated using permutational multivariate analysis of variance (PERMANOVA) and similarities percentage analysis (SIMPER). Univariate traits were analyzed using analysis of variance (ANOVA) and Tukey post-hoc tests. Additional details of the statistical analyses and software used are provided in the Supplemental Text.

## Results and discussion

### Which corals will survive chronic future ocean conditions?

**Figure 4:**
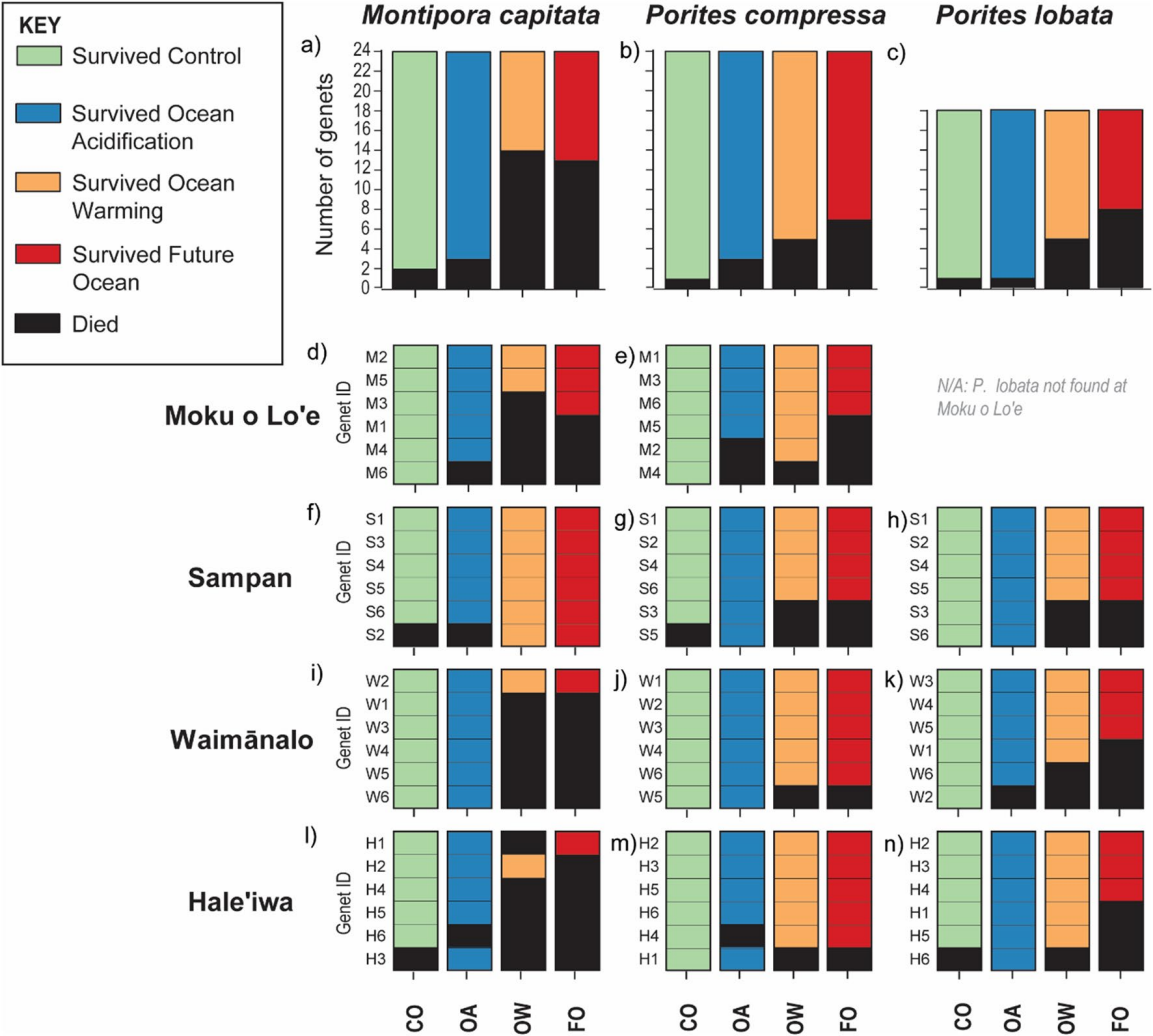
Survivorship of corals following 22 months exposure under control (CO, green), ocean acidification (OA, blue), ocean warming (OW, orange), or combined future ocean (FO, red) conditions for (**a**) *M. capitata* across sites, (**b**) *P. compressa* across site, (**c**) *P. lobata* across sites. Survivorship of the individual genets of each species collected from (**d**–**e**) Moku o Loʻe, (**f**–**h**) Sampan, (**i**–**k**) Waimānalo, and (**l**–**n**) Haleʻiwa.

Coral survivorship was influenced by temperature treatment and coral species (Table S5). Overall, 61% of corals exposed to elevated temperature survived, whereas 92% survived under ambient temperature conditions (Fig. 4). Across treatments, *Montipora capitata* had significantly lower survivorship relative to *Porites compressa* (67% and 83% respectively, Fig. 4). Under the combined future ocean treatment, 46% of *M. capitata*, 71% of *P. compressa*, and 56% of *P. lobata* genets survived (Fig. 4). This indicates that in the future, under prolonged exposure to elevated temperature and *p*CO_2_, reefs will suffer a dramatic decline in coral cover and loss of genotypic diversity. However, between 46–71% of genets of three of the most common species in Hawaiʻi were able to survive (Fig. 4a–c), and no extirpation (100% mortality of all genets) was observed for any species from any site (Fig. 4 d–n). However, survival alone is not sufficient to evaluate the health and longevity of corals under combined future ocean conditions. For example, if corals are unable to calcify at sufficient rates, then reefs will not be able to keep up with sea-level rise or provide habitat and structure necessary for ecosystem function^47^. Likewise, if corals are unable to reproduce, then their fitness will be compromised and they will not contribute to future generations (i.e., so called “zombie” corals^48^). Finally, if corals are not able to meet metabolic demands through photosynthesis and/or heterotrophy, then they will eventually deplete energy reserves and die. Evaluating how surviving coral holobionts physiologically cope with future ocean conditions can reveal the longer-term prognosis for their health and persistence, and can be used to improve the accuracy of future projections of coral bleaching^49^.

### How well do survivors cope with future ocean conditions?

**Figure 5:**
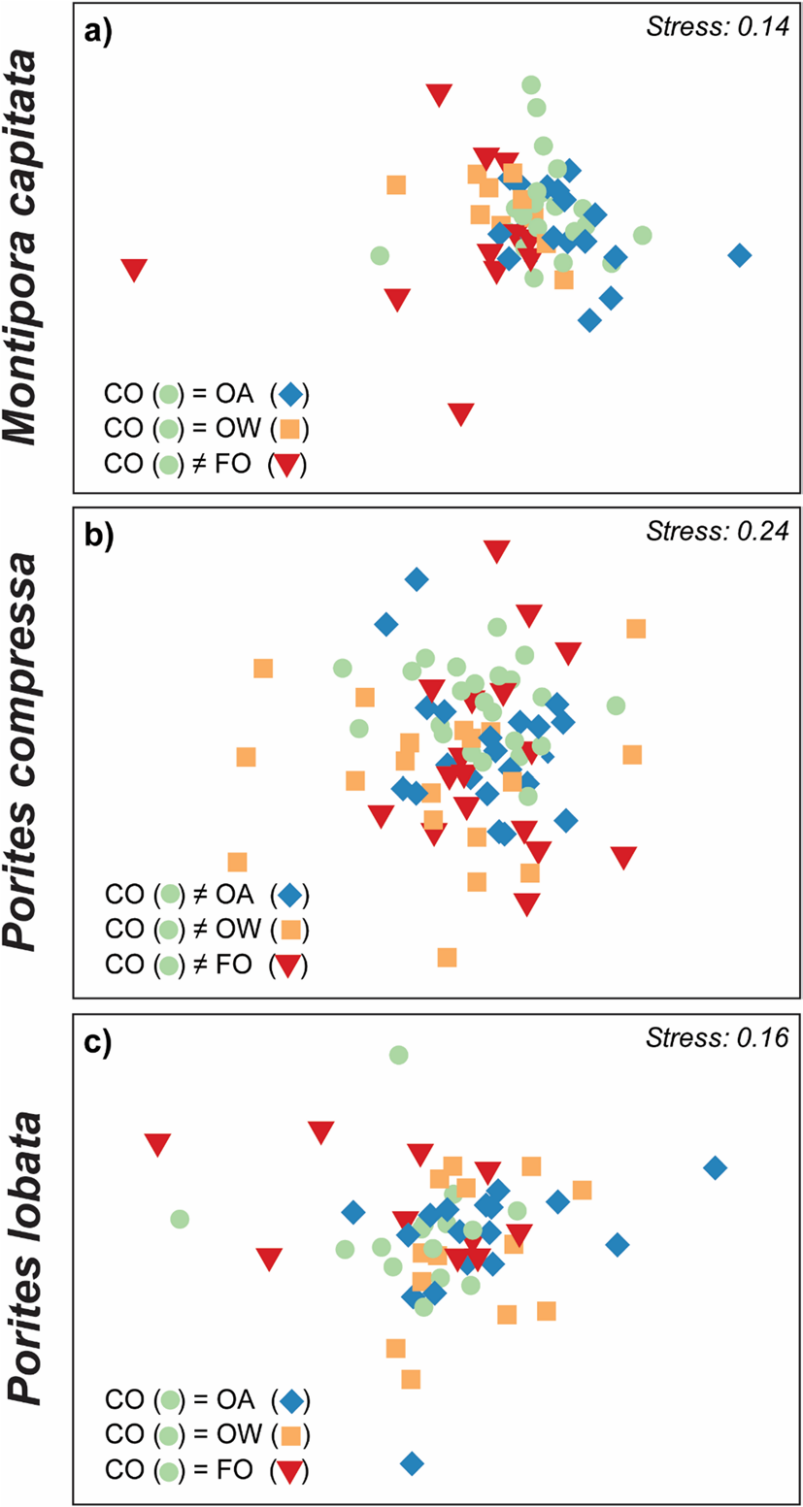
Nonparametric multidimensional scaling plots (NMDS) of coral physiological profiles for (**a**) *Montipora capitata*, (**b**) *Porites compressa*, and (**c**) *Porites lobata*. Data colors correspond to treatments: controls (CO, green circles), ocean acidification (OA, blue diamonds), ocean warming (OW, orange squares), or combined future ocean (FO, red triangles) treatments. Summary of pairwise PERMANOVA tests between CO and each of the treatments is shown in the bottom left of each panel. Additional statistical details in Table S7.

The physiological profiles of *Montipora capitata* survivors were primarily influenced by temperature, whereas the interaction between temperature and *p*CO_2_ was found to be significant for both species of *Porites* (Table S6). The physiological profiles of the surviving corals under combined future ocean conditions significantly differed from the controls of *Montipora capitata* and *Porites compressa* (Fig. 5a, b, Table S7a, b), but not *Porites lobata* (Fig. 5c, Table S7c).

**Figure 6:**
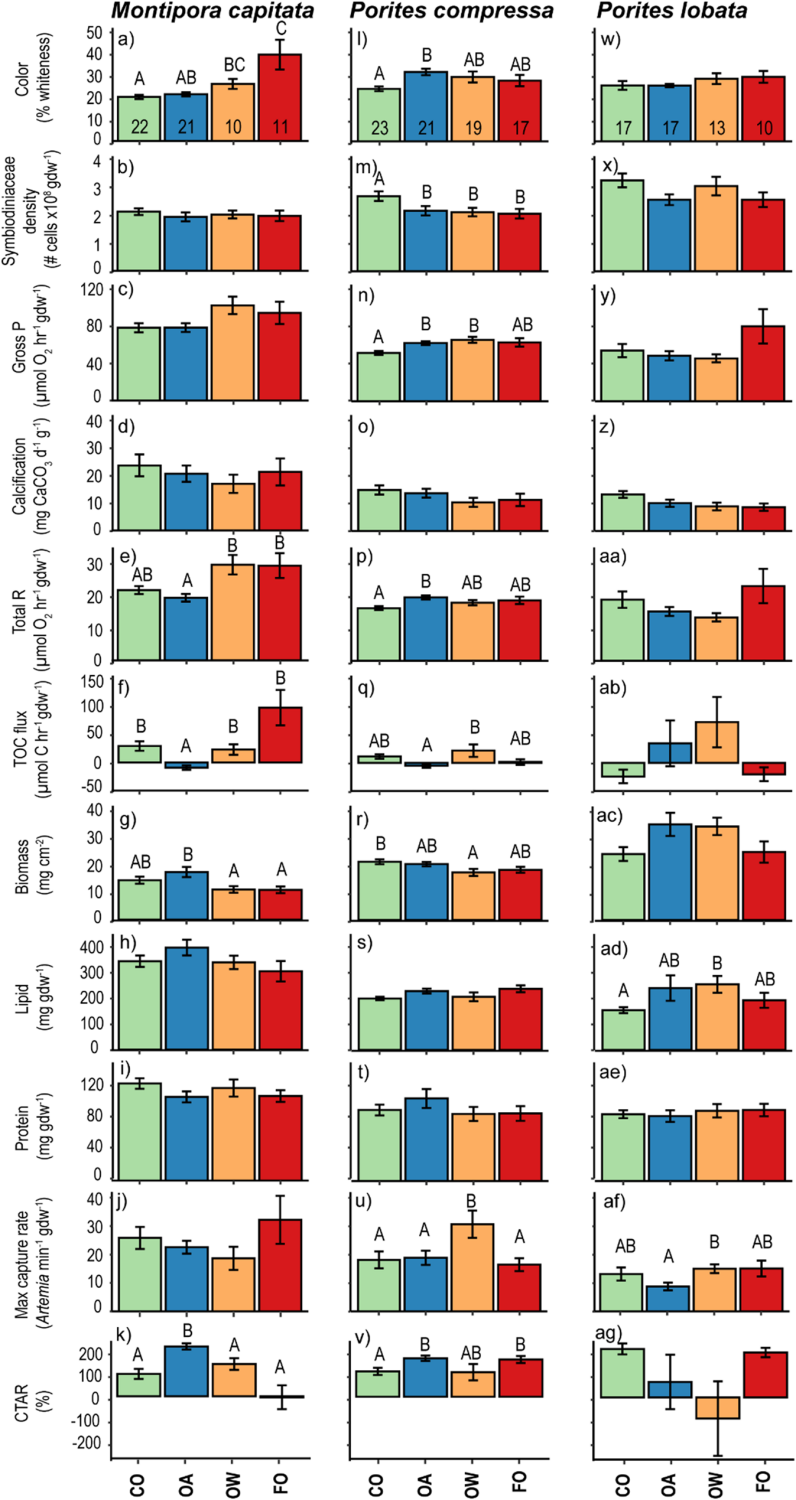
Mean (±1SE) of physiological traits in (**a-k**) *Montipora capitata*, (**l-v**) *Porites compressa*, and (**w-ag**) *Porites lobata* following 22-months exposure to control (CO, green), ocean acidification (OA, blue), ocean warming (OW, orange), or combined future ocean (FO, red) experimental treatment conditions. Uppercase letters above bars indicate the results of post-hoc tests when ANOVA was significant, whereby averages sharing letters did not significantly differ from each other. Sample sizes for each variable are shown within each bar in the top row and is the same for all panels below. Statistical details in Table S8. TOC = Total Organic Carbon Flux. CTAR = Contribution of the Total acquired fixed carbon relative to Animal Respiration.

For *M. capitata,* the dissimilarity between combined future ocean and control physiological profiles was driven primarily by color, TOC flux, and maximum feeding capacity (Table S7a), of which the first two were higher under combined future ocean conditions compared to control conditions (Fig. 6a, f, j). The 20% increase in whiteness in *M. capitata* was due to degraded chlorophyll pigments while maintaining Symbiodiniaceae cell density (Fig. 6a, b)—a well-documented mechanism of bleaching for this species^50^. Though photosynthesis and calcification rates were unaffected by treatment (Fig. 6c, d), respiration rates increased by 35% under the ocean warming and future ocean conditions suggesting a metabolic cost to exposure to elevated temperature (Fig. 6e). Corals can supplement their carbon budget by increasing heterotrophy and/or catabolizing their tissues and energy reserves (e.g.,^9,24,28,29^). However, we found that *M. capitata* experienced an average 236% increase in TOC fluxes under combined future ocean conditions compared with controls (Fig. 6f), resulting in dramatic fixed carbon losses and decreases in CTAR (Fig. 6k) that fell to an average of -4%—well below the necessary 100% level—all while maintaining lipids, protein, and biomass (Fig. 6g, h, i). This is clearly unsustainable. These patterns suggest that the inability to fully meet metabolic demand under combined future ocean conditions may explain the higher mortality rates observed in *M. capitata* relative to *P. compressa* (Table S5). However, it is important to consider that bleached *M. capitata* are capable of substantially upregulating zooplankton heterotrophy to meet > 100% of their metabolic demand on the reef where demersal zooplankton concentrations are abundant^28,51^. In this study, *M. capitata* under future ocean conditions had the highest maximum feeding capacity measured (Fig. 4j), but zooplankton were not provided to the corals in the evening when corals typically have their polyps extended and feed^52^. Hence, corals like *M. capitata* which rely on heterotrophic food sources to survive and recover from bleaching will likely perform better in situ than observed here and may even have higher survivorship on reefs with higher natural zooplankton concentrations.

For *Porites compressa*, photosynthesis, respiration, and Symbiodiniaceae density were the main drivers of separation between the holobiont physiological profiles of control and the surviving future ocean treatment group (Table S7b). Symbiodiniaceae density was 23% lower in the combined future ocean treatment than in the controls (Fig. 6m). At the same time, the percent whiteness of *P. compressa* did not differ between the controls and combined future ocean treatment ramets (Fig. 6l), suggesting an increase in chlorophyll concentration per Symbiodiniaceae cell. This photo-acclimatory response has been previously observed in *P. compressa*^50,51^ and likely allowed this species to maintain the fixed carbon acquired through photosynthesis relative to the control group (Fig. 6n, v). Though photosynthesis, respiration, and TOC fluxes were not significantly different between the future ocean and control corals (Fig. 6n, p, q), the net effect of the future ocean conditions on the sum of all carbon budget variables resulted in a significant increase in CTAR values from 110% in the controls to 162% in the future ocean treatment survivors (Fig. 6v). Thus, *P. compressa* met > 100% of their metabolic energy requirements under combined future ocean conditions allowing this species to maintain energy reserves and calcification rates (Fig. 6o, r–t,), and these responses likely contributed to the low mortality rates observed in this species (Fig. 4b). Maximum *Artemia* capture rate did not change under the future ocean treatment, but increased 69% in the ocean warming treatment relative to the controls (Fig. 6u).

Unlike the other two species, *P. lobata* survivors under future ocean conditions did not significantly differ in their physiological profile relative to the controls (Fig. 5c, Table S7c) and no differences were found between the control and combined future ocean treatment ramets for any of the ten phenotypic traits measured (Fig. 6w–ag). Thus, the capacity for survivors of this species to cope with prolonged exposure to elevated temperature and *p*CO_2_ cannot be explained by any of the phenotypic traits measured here. Interestingly, the composition of the microbiome of *P. lobata* does differ between the control and combined future ocean treatment survivors^53^ suggesting that the microbiome may be a key element in the survival and coping mechanism of this species. We suggest that changes in physiological profile which occur in response to the single stress of either increased *p*CO_2_ or elevated temperature are counteracted when combined, and thus help drive resilience among survivors of combined future ocean conditions. This is especially apparent with CTAR, where corals under either ocean acidification or ocean warming failed to meet 100% of metabolic demand but exceeded 200% of metabolic demand under combined future ocean conditions and maintained calcification and energy reserves (Fig. 6ag).

### Implications for future reefs

This is the longest ocean warming and acidification mesocosm experiment conducted on corals to date, and the only study to assess detailed holobiont physiological profiles following chronic, multiannual exposure to elevated temperature and *p*CO_2_ than include both diurnal and seasonal variability (Table S2). Our study sampled corals across a range of sites with differing environmental conditions, to help ensure that a representative sample of their phenotypic and genotypic diversity was included (Table S2). Therefore, this study provides insight into the capacity for corals to survive and identifies physiological traits that underly the capacity to cope with the future environmental change. We show that despite some mortality, at least two-thirds of *P. compressa* and *P. lobata* genets can survive and cope with future ocean conditions consistent with current commitments under the Paris Climate Agreement. While many survivors of *M. capitata* struggled to meet metabolic demand and cope, the lack of zooplankton supplementation may have unintentionally selected against this heterotrophically plastic species^28,51^ and unrealistically reduced survivorship. Nevertheless, we have shown that survivors of these three coral species are able to maintain calcification rates and tissue biomass, and maintain or increase lipid and protein energy reserves. In addition, both *Porites* coral species maintain or increase fixed carbon acquisition to meet or exceed their daily metabolic requirements. Thus, our finding provide hope that significant biological diversity within *Porites*, and several *M. capitata* genotypes, will persist this century provided atmospheric carbon dioxide levels are controlled within the commitments of the Paris Climate Agreement. Since *Porites* corals are ubiquitous throughout the world’s oceans, and *P. lobata* is a major reef builder in the Pacific, the resilience and persistence of *Porites* corals provides hope that some reef ecosystem function could be maintained globally.

### Ethics approval

Samples were collected under State of Hawaiʻi Department of Land & Natural Resources Division of Aquatic Resources Special Activity Permit (SAP) #2015-48.

## Supplementary Information

Supplementary Information.

## Data Availability

All data needed to evaluate the conclusions in the paper are present in the paper and/or the Supplementary Materials. The raw data analyzed for this study are deposited at BCO-DMO https://www.bco-dmo.org/project/546273.
