# Supplementary material for: Physiological acclimatization in Hawaiian corals following a 22-month shift in baseline seawater temperature and pH
Source: Sci Rep. 2022 Mar 10;12:3712. doi: 10.1038/s41598-022-06896-z (PMC8913750; doi:10.1038/s41598-022-06896-z)
Supplement: Supplementary file 1 — Supplementary Information. [file 41598_2022_6896_MOESM1_ESM.docx]

**SUPPLEMENTARY DOCUMENT**

Physiological acclimatization in Hawaiian corals following a 22-month shift in baseline seawater temperature and pH

**Authors:** Rowan H. McLachlan^1,2*^, James T. Price^1^, Agustí Muñoz-Garcia^3^, Noah L. Weisleder^4^, Stephen J. Levas^5^, Christopher P. Jury^6^, Robert J. Toonen^6^, Andréa G. Grottoli^1^

**Affiliations:**

^1^ School of Earth Sciences, The Ohio State University, 125 South Oval Mall, Columbus, OH 43210

^2^ Department of Microbiology, Oregon State University, 2820 SW Campus Way, Corvallis, OR 97331

^3^ Department of Evolution, Ecology and Organismal Biology, The Ohio State University at Mansfield, 1760 University Dr., Mansfield, OH 44906

^4^ Department of Physiology and Cell Biology, The Ohio State University, 473 West 12th Avenue, Columbus, OH 43210

^5^ Geography, Geology and Environmental Science, University of Wisconsin – Whitewater, 800 W. Main Street, Whitewater, WI 53190

^6^ Hawaiʻi Institute of Marine Biology, University of Hawaiʻi at Mānoa, 46-007 Lilipuna Road, Kāneʻohe, HI 96744

^*^ **Co-corresponding authors:** [mclachlan.8@osu.edu](mailto:mclachlan.8@osu.edu); [grottoli.1@osu.edu](mailto:grottoli.1@osu.edu)

**Supplementary Text**

***Mesocosm experiment***

The experiment consisted of four treatments (n = 10 mesocosms per treatment) as follows: control (present-day temperature with present-day *p*CO*_2_*), ocean acidification (present-day temperature with +350 μatm *p*CO*_2_*), ocean warming (+2 ℃ with present-day *p*CO*_2_*), and combined future ocean conditions (+2 ℃ with +350 μatm *p*CO*_2_*). The temperature and *p*CO_2_ levels are consistent with current commitments under the Paris Climate Agreement (*33*). The ramets of *M. capitata*, *P. compressa*, and *P. lobata* were distributed among the 40 outdoor flow-through mesocosm tanks (70 L, 50 x 50 x 30 cm) at HIMB such that one ramet per genet was present within each of the four treatment conditions. Starting on 1 February 2016, temperature and *p*CO_2_ were adjusted gradually over 20 days to minimize the likelihood of shocking the mesocosm communities. As the incoming waters from Kāneʻohe Bay are naturally slightly warmer and more acidic than other nearby reefs (*50*–*53*), the seawater being delivered to the control and ocean acidification treatment mesocosm tanks was chilled by 0.5 °C after 10 and 20 days while the seawater delivered to the ocean warming and combined future ocean treatment mesocosms was warmed by 0.5 °C after 10 and 20 days, then maintained at these offsets for the remainder of the study. At the same time, pH of the seawater being delivered to the ocean acidification and combined future ocean treatments was decreased by 0.05 units while the pH of the seawater being delivered to the control and ocean warming treatment mesocosm tanks was increased by 0.05 units after 10 days and again after 20 days then maintained at the offsets for the remainder of the study. Corals were maintained under experimental conditions for 22 months from 20 February 2016 to 13 December 2017 for a total of 662 days (Fig. S1). Salinity, temperature, *p*CO_2_, and pH were measured at mid-day in each mesocosm once weekly and treatment average weekly values (± 1SD) were plotted (Fig S2). This is a long-term experiment as defined by (*34*, *35*) and the longest dual stress (i.e., combined ocean warming and acidification) experiment on corals to date (Table S2).

The mesocosms were designed to mimic the natural reef environment as closely as possible. Each mesocosm contained fragments from the eight most dominant reef-building coral species in Hawaiʻi (*Montipora capitata*, *Montipora flabellata*, *Montipora patula*, *Porites compressa*, *Porites lobata*, *Porites evermanni*, *Pocillopora* *meandrina*, and *Pocillopora* *acuta*) (*54*, *55*), a layer of sand and carbonate rubble, a juvenile Convict tang (*Acanthurus triostegus*), and a juvenile Threadfin butterflyfish (*Chaetodon auriga*). Both fishes are generalist grazers, where the convict tang feeds on benthic algae and the butterflyfish feeds on non-coral invertebrates. They were at representative fish biomass densities for Hawaiian reefs (*56*), and together provide the essential functional role of herbivory and predation within the mesocosm communities. The flow-through mesocosms received unfiltered seawater pumped directly from the neighboring reef within Kāneʻohe Bay. Seawater was initially pumped into one of eight header tanks (two per treatment) within which temperature and pH were manipulated and then subsequently directed into the mesocosms such that each header tank supplied five mesocosms. Coral fragments were not directly fed but had access to dissolved and particulate organic matter from the reef-derived seawater and from daily feeding of the fish who were supplied ~3 g wet weight of frozen adult mysid or *Artemia* brine shrimp under flow-through conditions, thereby provisioning the fish and mesocosm communities with allochthonous (i.e., non-local, imported) non-living zooplankton at a rate similar to that measured in nature (*57*).

The coral fragments grew faster than expected during the experimental period and therefore space within each mesocosm became limited near the end of the experiment. In order to prolong the experiment, three of the eight coral species (*Montipora flabellata, Pocillopora meandrina, and Porites evermanni*) were transferred into a secondary mesocosm system at HIMB in the last month of this study on 18 November 2017, thereby increasing the available space to all remaining coral fragments. None of the *M. capitata, P. compressa* and *P. lobata* corals showed any obvious adverse reactions to having *M. flabellata, P. meandrina,* and *P. evermanni* removed from the primary mesocosm tanks.

Coral fragments were photographed for surface area and ramet whiteness analysis, and buoyant weighed on the weeks of 20 March 2016 and 27 November 2017 corresponding to one month after the target temperature and pH conditions were reached and the end of the experimental period, respectively (Fig. S1). During the last 20 days of the experimental period (23 November–13 December 2017) the following live physiological measurements were conducted on all surviving coral ramets: photosynthesis, respiration, total organic carbon flux, and maximum *Artemia* feeding capacity (Fig. S1). Then, all surviving coral fragments were sacrificed by freezing at -20 ℃. Samples were transported on dry ice to the Ohio State University (OH, USA) where they were stored at -80 ℃ awaiting further analyses of biomass, lipids, proteins, Symbiodiniaceae density, and surface area according to methods published in protocols.io (*36*–*40*). Additional details below.

***Laboratory Methods***

Coral color and surface area

Photographs of corals were taken from six different angles next to a scale bar and white reference card. Using ImageJ software, coral whiteness was assessed via photographic image analysis using the greyscale model to quantify the bleaching appearance (*17*). The percent whiteness was used as a proxy for bleaching intensity of corals because it is known to be highly correlated with chlorophyll *a* and Symbiodiniaceae density (*16*, *17*). Coral surface area was estimated from photographs using the geometric method (Naumann et al. 2009) for which a detailed protocol is described in McLachlan and Grottoli (2021).

Calcification

Calcification rate was determined using the buoyant weight technique (*59*). Daily calcification rates were calculated as the difference between initial and final weights, divided by the respective number of days elapsed, and normalized to the initial weight of the skeleton. Equations for calcification rate are shown in Table S4a.

Photosynthesis, respiration, and total organic carbon flux

Maximal photosynthesis and day and night respiration rates were measured via changes in dissolved oxygen for each individual coral ramet (*23*) at respective treatment seawater temperatures and *p*CO_2_ levels, and normalized to ash-free dry weight (AFDW). Total respiration was calculated by summing day and night respiration rates multiplied by the respective number of hours per day (i.e., 11 daytime hours and 13 nighttime hours at the time of our measurements in Hawaiʻi). Photosynthesis and respiration rates were corrected for any change in seawater oxygen concentration due to microorganism respiration which occurred in a seawater blank control chamber. Equations for photosynthesis and respiration rate are shown in Table S4b. Due to the high growth rates exhibited by many of the corals during the experiment, they were far too large to fit into the original respirometry chambers by the end of the study. Therefore, a smaller sub-ramet was cut from each ramet of *M. capitata* and *P. compressa* using a band saw with a diamond-coated blade and each sub-ramet was mounted on a labelled ceramic plug using cyanoacrylate gel for respirometry. The *P. lobata* grew primarily horizontally rather than vertically and thus were not cut prior to live physiological analyses and instead new wider respirometry chambers were constructed to accommodate them.

Total organic carbon (TOC) water samples were collected following night respiration incubations because coral feeding is known to occur primarily after dusk, using methods adapted from Levas et al. (2015). Following night respiration incubations, the water level in respirometry bins was lowered to expose the top of coral incubation chambers. While wearing nitrile gloves, lids were removed and corals returned to experimental tanks. A 30 ml water sample was removed from each chamber using a new disposable 10 ml pipet, filtered through a 55 µm Nitex mesh, and collected in a pre-cleaned 50 ml Nalgene bottle. All water samples were acidified within 10 minutes of collection using 1 ml of 1.2 M hydrochloric acid (ACS Reagent Grade). TOC concentrations were determined using high-temperature catalytic oxidation using a Shimadzu model TOC-L analyzer, were corrected for the volume of water in respirometry chambers, and divided by the incubation duration to obtain the flux values per hour. The TOC value of the non-coral containing control chambers were subtracted from coral chamber TOC values. Corrected TOC fluxes were normalized to AFDW. Equations for TOC flux are shown in Table S4c.

Carbon budget

The carbon budget of each coral ramet was calculated to determine the proportionate contribution of photosynthesis and heterotrophy to total metabolic demand (i.e., respiration). Photosynthesis and total respiration rates were used to calculate the percent Contribution of Zooxanthellae (i.e., Symbiodiniaceae) to Animal Respiration (CZAR) (*41*), while total respiration and nighttime TOC flux rates were used to calculate the percent Contribution of Heterotrophy from TOC to Animal Respiration (CHAR_TOC_) (*42*). *Artemia* feeding capacity was not used to calculate CHAR_zoop_ as the *Artemia* concentrations were not representative of reef zooplankton densities or mesocosm zooplankton densities. The Contribution of the Total acquired fixed carbon relative to Animal Respiration (CTAR) (*27*) was calculated as the sum of CZAR and CHAR_TOC_. However, we acknowledge that this is likely an underestimate of CTAR as it does not account for heterotrophic carbon derived from zooplankton nor any potential gains or losses in CHAR_TOC_ that may have occurred during the day. Carbon budget equations are shown in Table S4d.

Maximum Artemia capture rate

The maximum *Artemia* capture rate of corals was assessed using methods adapted from (*60*). Briefly, corals were placed upon a small plastic stand in individual 500 ml glass beakers filled with seawater from their respective treatments. Beakers were placed on top of a magnetic stir plate (200 RPM) within a 100 L water bath maintained at the desired experimental temperature and placed in front of a window. Conducting feeding measurements under natural moonlight has been observed to increase polyp expansion and feeding behavior (Grottoli pers. obs.). Corals were placed in the feeding beakers one hour before sunset to ensure polyp expansion. Approximately 30 min after sundown, a concentrated solution of 2-day old *Artemia salina* nauplii was added to each beaker at an average concentration of 3000–3500 *Artemia* L^-1^. This concentration is much higher than *in situ* zooplankton concentrations, but was chosen to assess the maximum zooplankton capture rate of corals, as feeding rate in known to increase with prey concentration (*61*, *62*). Five 10 ml subsamples were removed from each beaker using a 10 ml glass pipette after 2 and 40 minutes and the number of *Artemia* in the pipette was immediately counted under a light microscope. The counted *Artemia* solution was returned to the beaker within 30 seconds of its initial removal. After the final count, corals were removed from beakers and returned to their experimental tanks. The maximum *Artemia* feeding rate was calculated as the difference between average initial and end concentrations of *Artemia*, divided by the volume of water in the feeding beaker and the duration of the feeding trial. Capture rates were corrected for any change in *Artemia* concentration which occurred in a control beaker without coral. Maximum *Artemia* capture rates were normalized to AFDW. Equations for maximum *Artemia* capture rate are shown in Table S4e.

Biomass, lipid, protein, and Symbiodiniaceae density

Frozen coral fragments were ground into a homogenous paste using a chilled mortar and pestle and partitioned using methods described in McLachlan, Dobson & Grottoli (2020). Between 0.5–1 g of ground material was partitioned for analyses of total biomass ash-free dry weight, total soluble lipid, total soluble protein (henceforth referred to as biomass, lipid, and protein, respectively), and Symbiodiniaceae density based on pre-determined needs for each analysis. Briefly, biomass was quantified by drying ground coral subsamples to a constant weight (60 ℃ for 24 hr) and burning it (450 ℃ for 6 hr) according to protocol methods detailed in McLachlan, Dobson & Grottoli (2020). Lipids were extracted using 2:1 chloroform methanol using methods modified from Rodrigues and Grottoli (2007) and the protocol detailed in McLachlan, Muñoz-Garcia & Grottoli (2020). Protein was quantified using the Bradford method (Bradford 1976) with protocol details in McLachlan et al., (2020a). Symbiodiniaceae density was quantified by counting the number of cells in four replicate (4 µL) subsamples using a Countess™ II FL Automated Cell Counter which is detailed in McLachlan, Juracka & Grottoli (2020). Coral biomass was normalized to surface area (*40*), and lipid, protein, and Symbiodiniaceae density were normalized to AFDW to facilitate comparison among species of varying morphologies with different surface-area-to-volume ratios (*63*).

***Data analysis***

To test for the effects of species temperature, and *p*CO_2_ on survivorship, survivorship data were analyzed using a generalized linear model with a binomial error distribution with species, temperature, and *p*CO_2_ as fixed effects. Pairwise post-hoc slice-tests of main effects were performed using estimated marginal means.

To identify the physiological mechanisms underlying the ability of survivors of each species to cope (or not cope) with future ocean conditions, multivariate statistical analyses were performed using ten phenotypic traits simultaneously: color, Symbiodiniaceae density, gross photosynthesis, calcification, total respiration, TOC flux, biomass, lipid, protein and, maximum *Artemia* capture rate. These ten physiological traits (henceforth referred to as the holobiont physiological profile of corals) were standardized prior to the construction of Euclidean distance resemblance matrices. Data were first visualized using non-parametric multidimensional scaling (NMDS) plots for each species. The effects of treatment on coral physiological profiles were investigated using nested permutational multivariate analysis of variance (PERMANOVA) with temperature and *p*CO_2_ as fixed effects and header and mesocosm tank as random effects. Where significant temperature by *p*CO_2_ interactions were identified, we conducted pseudo-post hoc analysis by one-way PERMANOVA pairwise tests with treatment as a fixed effect (4 levels: control, ocean acidification, ocean warming, combined future ocean). Similarities percentage analysis (SIMPER) was used to identify which underlying phenotypic traits were driving the largest proportion of the variance among treatment groups.

To explore how each phenotypic trait in the physiological profiles was changing in response to treatments, one-way analysis of variance (ANOVA) tests were performed for each physiological trait for each species where treatment was a fixed effect. Tukey posthoc tests with Benjamini-Hochberg correction for multiple comparisons were performed to determine which treatments significantly differed from each other. When ANOVA assumptions could not be met by transforming data, Kruskal Wallis one-way analysis of variance and Dunn’s posthoc tests with Benjamini-Hochberg correction for multiple comparisons were used. While this is technically a two-way system, analyzing the data that way does not address our hypotheses nor help us identify relevant patterns in the data. Thus, one-way ANOVAS were used. All univariate and multivariate statistics were performed using R v3.6.2 (*64*) and PERMANOVA + for PRIMER v6 (*65*), respectively.

## References

1. T. P. Hughes, M. L. Barnes, D. R. Bellwood, J. E. Cinner, G. S. Cumming, J. B. C. Jackson, J. Kleypas, I. A. Van De Leemput, J. M. Lough, T. H. Morrison, S. R. Palumbi, E. H. Van Nes, M. Scheffer, Coral reefs in the Anthropocene. *Nature*. **546**, 82–90 (2017).

2. T. P. Hughes, J. T. Kerry, M. Álvarez-Noriega, J. G. Álvarez-Romero, K. D. Anderson, A. H. Baird, R. C. Babcock, M. Beger, D. R. Bellwood, R. Berkelmans, T. C. Bridge, I. R. Butler, M. Byrne, N. E. Cantin, S. Comeau, S. R. Connolly, G. S. Cumming, S. J. Dalton, G. Diaz-Pulido, C. M. Eakin, W. F. Figueira, J. P. Gilmour, H. B. Harrison, S. F. Heron, A. S. Hoey, J. P. A. Hobbs, M. O. Hoogenboom, E. V. Kennedy, C. Y. Kuo, J. M. Lough, R. J. Lowe, G. Liu, M. T. McCulloch, H. A. Malcolm, M. J. McWilliam, J. M. Pandolfi, R. J. Pears, M. S. Pratchett, V. Schoepf, T. Simpson, W. J. Skirving, B. Sommer, G. Torda, D. R. Wachenfeld, B. L. Willis, S. K. Wilson, Global warming and recurrent mass bleaching of corals. *Nature*. **543**, 373–377 (2017).

3. T. P. Hughes, K. D. Anderson, S. R. Connolly, S. F. Heron, J. T. Kerry, J. M. Lough, A. H. Baird, J. K. Baum, M. L. Berumen, T. C. Bridge, D. C. Claar, C. M. Eakin, J. P. Gilmour, N. A. J. Graham, H. Harrison, J. P. A. Hobbs, A. S. Hoey, M. O. Hoogenboom, R. J. Lowe, M. T. McCulloch, J. M. Pandolfi, M. Pratchett, V. Schoepf, G. Torda, S. K. Wilson, Spatial and temporal patterns of mass bleaching of corals in the Anthropocene. *Science (80-. ).* **359**, 80–83 (2018).

4. C. M. Eakin, H. P. A. Sweatman, R. E. Brainard, The 2014–2017 global-scale coral bleaching event: insights and impacts. *Coral Reefs*. **38**, 539–545 (2019).

5. Glynn, Coral reef bleaching: facts, hypotheses and implications. *Glob. Chang. Biol.* **2**, 495–509 (1996).

6. B. E. Brown, Coral bleaching: Causes and consequences. *Coral Reefs*. **16**, 129–138 (1997).

7. J. A. Maynard, R. Van Hooidonk, C. M. Eakin, M. Puotinen, M. Garren, G. Williams, S. F. Heron, J. Lamb, E. Weil, B. L. Willis, C. D. Harvell, Projections of climate conditions that increase coral disease susceptibility and pathogen abundance and virulence. *Nat. Clim. Chang.* **5**, 688–694 (2015).

8. T. P. Hughes, J. T. Kerry, A. H. Baird, S. R. Connolly, A. Dietzel, C. M. Eakin, S. F. Heron, A. S. Hoey, M. O. Hoogenboom, G. Liu, M. J. McWilliam, R. J. Pears, M. S. Pratchett, W. J. Skirving, J. S. Stella, G. Torda, Global warming transforms coral reef assemblages. *Nature*. **556**, 492–496 (2018).

9. K. R. N. Anthony, D. I. Kline, G. Diaz-Pulido, S. Dove, O. Hoegh-Guldberg, Ocean acidification causes bleaching and productivity loss in coral reef builders. *Proc. Natl. Acad. Sci. U. S. A.* **105**, 17442–17446 (2008).

10. H. Huang, X. C. Yuan, W. J. Cai, C. L. Zhang, X. Li, S. Liu, Positive and negative responses of coral calcification to elevated pCO2: Case studies of two coral species and the implications of their responses. *Mar. Ecol. Prog. Ser.* **502**, 145–156 (2014).

11. K. D. Hoadley, D. T. Pettay, A. G. Grottoli, W. J. Cai, T. F. Melman, V. Schoepf, X. Hu, Q. Li, H. Xu, Y. Wang, Y. Matsui, J. H. Baumann, M. E. Warner, Physiological response to elevated temperature and pCO2 varies across four Pacific coral species: Understanding the unique host+symbiont response. *Sci. Rep.* **5**, 1–15 (2015).

12. V. Schoepf, A. G. Grottoli, M. E. Warner, W. J. Cai, T. F. Melman, K. D. Hoadley, D. T. Pettay, X. Hu, Q. Li, H. Xu, Y. Wang, Y. Matsui, J. H. Baumann, Coral energy reserves and calcification in a high-CO2 world at two temperatures. *PLoS One*. **8**, e75049 (2013).

13. IPCC, in *IPCC Special Report on the Ocean and Cryosphere in a Changing Climate*, H.-O. Pörtner, D. C. Roberts, V. Masson-Delmotte, P. Zhai, M. Tignor, E. Poloczanska, K. Mintenbeck, A. Alegría, M. Nicolai, A. Okem, J. Petzold, B. Rama, N. M. Weyer, Eds. (Cambridge University Press, Cambridge, United Kingdom and New York, NY, USA, 2019), pp. 1–36.

14. K. D. Bahr, P. L. Jokiel, K. S. Rodgers, Relative sensitivity of five Hawaiian coral species to high temperature under high-pCO2 conditions. *Coral Reefs*. **35**, 729–738 (2016).

15. S. G. Dove, K. T. Brown, A. Van Den Heuvel, A. Chai, O. Hoegh-Guldberg, Ocean warming and acidification uncouple calcification from calcifier biomass which accelerates coral reef decline. *Commun. Earth Environ.* **1**, 1–9 (2020).

16. M. H. Chow, R. H. L. Tsang, E. K. Y. Lam, P. O. Ang, Quantifying the degree of coral bleaching using digital photographic technique. *J. Exp. Mar. Bio. Ecol.* **479**, 60–68 (2016).

17. C. Amid, M. Olstedt, J. S. Gunnarsson, H. Le Lan, H. Tran Thi Minh, P. J. Van den Brink, M. Hellström, M. Tedengren, Additive effects of the herbicide glyphosate and elevated temperature on the branched coral Acropora formosa in Nha Trang, Vietnam. *Environ. Sci. Pollut. Res.* **25**, 13360–13372 (2018).

18. K. R. N. Anthony, S. R. Connolly, B. L. Willis, Comparative analysis of energy allocation to tissue and skeletal growth in corals. *Limnol. Oceanogr.* **47**, 1417–1429 (2002).

19. P. J. Edmunds, P. Spencer Davies, An energy budget for Porites porites (Scleractinia). *Mar. Biol.* **92**, 339–347 (1986).

20. J. S. Stimson, Location, quantity and rate of change in quantity of lipids in tissue of Hawaiian hermatypic corals. *Bull. Mar. Sci.* **41**, 889–904 (1987).

21. A. D. Harland, J. C. Navarro, P. Spencer Davies, L. M. Fixter, Lipids of some Caribbean and Red Sea corals: total lipid, wax esters, triglycerides and fatty acids. *Mar. Biol.* **117**, 113–117 (1993).

22. A. G. Grottoli, D. Tchernov, G. Winters, Physiological and biogeochemical responses of super-corals to thermal stress from the northern gulf of Aqaba, Red Sea. *Front. Mar. Sci.* **4**, 1–12 (2017).

23. L. J. Rodrigues, A. G. Grottoli, Energy reserves and metabolism as indicators of coral recovery from bleaching. *Limnol. Oceanogr.* **52**, 1874–1882 (2007).

24. K. R. N. Anthony, M. O. Hoogenboom, J. A. Maynard, A. G. Grottoli, R. Middlebrook, Energetics approach to predicting mortality risk from environmental stress: A case study of coral bleaching. *Funct. Ecol.* **23**, 539–550 (2009).

25. J. H. Baumann, A. G. Grottoli, A. D. Hughes, Y. Matsui, Photoautotrophic and heterotrophic carbon in bleached and non-bleached coral lipid acquisition and storage. *J. Exp. Mar. Bio. Ecol.* **461**, 469–478 (2014).

26. A. D. Hughes, A. G. Grottoli, Heterotrophic compensation: A possible mechanism for resilience of coral reefs to global warming or a sign of prolonged stress? *PLoS One*. **8**, 1–10 (2013).

27. A. G. Grottoli, M. E. Warner, S. J. Levas, M. D. Aschaffenburg, V. Schoepf, M. McGinley, J. H. Baumann, Y. Matsui, The cumulative impact of annual coral bleaching can turn some coral species winners into losers. *Glob. Chang. Biol.* **20**, 3823–3833 (2014).

28. A. G. Grottoli, L. J. Rodrigues, J. E. Palardy, Heterotrophic plasticity and resilience in bleached corals. *Nature*. **440**, 1186–1189 (2006).

29. S. J. Levas, A. G. Grottoli, V. Schoepf, M. D. Aschaffenburg, J. H. Baumann, J. E. Bauer, M. E. Warner, Can heterotrophic uptake of dissolved organic carbon and zooplankton mitigate carbon budget deficits in annually bleached corals? *Coral Reefs*. **35**, 495–506 (2016).

30. G. T. Concepcion, N. R. Polato, I. B. Baums, R. J. Toonen, Development of microsatellite markers from four Hawaiian corals: Acropora cytherea, Fungia scutaria, Montipora capitata and Porites lobata. *Conserv. Genet. Resour.* **2**, 11–15 (2010).

31. K. D. Gorospe, S. A. Karl, Genetic relatedness does not retain spatial pattern across multiple spatial scales: Dispersal and colonization in the coral, Pocillopora damicornis. *Mol. Ecol.* **22**, 3721–3736 (2013).

32. K. D. Bahr, T. Tran, C. P. Jury, R. J. Toonen, Abundance, size, and survival of recruits of the reef coral Pocillopora acuta under ocean warming and acidification. *PLoS One*. **15**, 1–13 (2020).

33. J. Rogelj, M. Den Elzen, N. Höhne, T. Fransen, H. Fekete, H. Winkler, R. Schaeffer, F. Sha, K. Riahi, M. Meinshausen, Paris Agreement climate proposals need a boost to keep warming well below 2 °c. *Nature*. **534**, 631–639 (2016).

34. R. H. McLachlan, J. T. Price, S. L. Solomon, A. G. Grottoli, Thirty years of coral heat-stress experiments: a review of methods. *Coral Reefs*. **39**, 885–902 (2020).

35. A. G. Grottoli, R. J. Toonen, R. van Woesik, R. Vega Thurber, M. E. Warner, R. H. McLachlan, J. T. Price, K. D. Bahr, I. B. Baums, K. D. Castillo, M. A. Coffroth, R. Cunning, K. L. Dobson, M. J. Donahue, J. L. Hench, R. Iglesias-Prieto, D. W. Kemp, C. D. Kenkel, D. I. Kline, I. B. Kuffner, J. L. Matthews, A. B. Mayfield, J. L. Padilla-Gamiño, S. Palumbi, C. R. Voolstra, V. M. Weis, H. C. Wu, Increasing comparability among coral bleaching experiments. *Ecol. Appl.* **31**, e02262 (2021).

36. R. H. McLachlan, K. L. Dobson, A. G. Grottoli, Microplate Assay for Quantification of Soluble Protein in Ground Coral Samples. *Protocols.io* (2020). doi:10.17504/protocols.io.bdyai7se.

37. R. H. McLachlan, A. Muñoz-Garcia, A. G. Grottoli, Extraction of Total Soluble Lipid from Ground Coral Samples. *Protocols.io* (2020). doi:10.17504/protocols.io.bc4qiyvw.

38. R. H. McLachlan, J. T. Price, K. L. Dobson, N. Weisleder, A. G. Grottoli, Microplate Assay for Quantification of Soluble Protein in Ground Coral Samples. *Protocols.io* (2020). doi:10.17504/ protocols.io.bdc8i2zw.

39. R. H. McLachlan, C. Juracka, A. G. Grottoli, Symbiodiniaceae Enumeration in Ground Coral Samples Using Countess™ II FL Automated Cell Counter. *Protocols.io* (2020). doi:10.17504/ protocols.io.bdc5i2y6.

40. R. H. McLachlan, A. G. Grottoli, Geometric Method for Estimating Coral Surface Area Using Image Analysis. *Protocols.io* (2021). https://doi.org/10.17504/protocols.io.bpxcmpiw

41. L. Muscatine, L. R. McCloskey, R. E. Marian, Estimating the daily contribution of carbon from zooxanthellae to coral animal respiration. *Limnol. Oceanogr.* **26**, 601–611 (1981).

42. S. J. Levas, A. G. Grottoli, M. E. Warner, W. J. Cai, J. E. Bauer, V. Schoepf, J. H. Baumann, Y. Matsui, C. Gearing, T. F. Melman, K. D. Hoadley, D. T. Pettay, X. Hu, Q. Li, H. Xu, Y. Wang, Organic carbon fluxes mediated by corals at elevated pCO2 and temperature. *Mar. Ecol. Prog. Ser.* **519**, 153–164 (2015).

43. C. T. Perry, L. Alvarez-Filip, N. A. J. Graham, P. J. Mumby, S. K. Wilson, P. S. Kench, D. P. Manzello, K. M. Morgan, A. B. A. Slangen, D. P. Thomson, F. Januchowski-Hartley, S. G. Smithers, R. S. Steneck, R. Carlton, E. N. Edinger, I. C. Enochs, N. Estrada-Saldívar, M. D. E. Haywood, G. Kolodziej, G. N. Murphy, E. Pérez-Cervantes, A. Suchley, L. Valentino, R. Boenish, M. Wilson, C. MacDonald, Loss of coral reef growth capacity to track future increases in sea level. *Nature*. **558**, 396–400 (2018).

44. C. M. Woodley, A. Burnett, C. A. Downs, “Epidemiological Assessment of Reproductive Condition of ESA Priority Coral” (2013).

45. C. A. Logan, J. P. Dunne, C. M. Eakin, S. D. Donner, Incorporating adaptive responses into future projections of coral bleaching. *Glob. Chang. Biol.* **20**, 125–139 (2014).

46. L. J. Rodrigues, A. G. Grottoli, M. P. Lesser, Long-term changes in the chlorophyll fluorescence of bleached and recovering corals from Hawaii. *J. Exp. Biol.* **211**, 2502–2509 (2008).

47. V. Schoepf, A. G. Grottoli, S. J. Levas, M. D. Aschaffenburg, J. H. Baumann, Y. Matsui, M. E. Warner, Annual coral bleaching and the long-term recovery capacity of coral. *Proc. R. Soc. B Biol. Sci.* **282**, e20151887 (2015).

48. J. E. Palardy, L. J. Rodrigues, A. G. Grottoli, The importance of zooplankton to the daily metabolic carbon requirements of healthy and bleached corals at two depths. *J. Exp. Mar. Bio. Ecol.* **367**, 180–188 (2008).

49. F. Houlbrèque, C. Ferrier-Pagès, Heterotrophy in tropical scleractinian corals. *Biol. Rev.* **84**, 1–17 (2009).

50. J. T. Price, thesis, The Ohio State University (2020).

51. C. P. Jury, F. I. M. Thomas, M. J. Atkinson, R. J. Toonen, Buffer capacity, ecosystem feedbacks, and seawater chemistry under global change. *Water (Switzerland)*. **5**, 1303–1325 (2013).

52. C. P. Jury, R. J. Toonen, Adaptive responses and local stressor mitigation drive coral resilience in warmer, more acidic oceans. *Proc. R. Soc. B Biol. Sci.* **286**, 20190614 (2019).

53. R. H. McLachlan, J. T. Price, A. Muñoz-Garcia, N. L. Weisleder, C. P. Jury, R. J. Toonen, A. G. Grottoli, Environmental gradients drive physiological variation in Hawaiian corals. *Coral Reefs* (2021), doi:10.1007/s00338-021-02140-8.

54. K. S. Rodgers, P. L. Jokiel, E. K. Brown, S. Hau, R. Sparks, Over a decade of change in spatial and temporal dynamics of Hawaiian coral reef communities. *Pacific Sci.* **69**, 1–13 (2015).

55. E. C. Franklin, P. L. Jokiel, M. J. Donahue, Predictive modeling of coral distribution and abundance in the Hawaiian Islands. *Mar. Ecol. Prog. Ser.* **481**, 121–132 (2013).

56. K. D. Gorospe, M. J. Donahue, A. Heenan, J. M. Gove, I. D. Williams, R. E. Brainard, Local biomass baselines and the recovery potential for Hawaiian coral reef fish communities. *Front. Mar. Sci.* **5**, 1–13 (2018).

57. W. M. Hamner, M. S. Jones, J. H. Carleton, I. R. Hauri, D. M. Williams, Currents on a Windward Reef Face. *Bull. Mar. Sci.* **42**, 459–479 (1988).

58. M. S. Naumann, W. Niggl, C. Laforsch, C. Glaser, C. Wild, Coral surface area quantification-evaluation of established techniques by comparison with computer tomography. *Coral Reefs*. **28**, 109–117 (2009).

59. P. L. Jokiel, J. E. Maragos, L. Franzisket, in *Coral Reefs: Research Methods* (1978; https://www.researchgate.net/profile/Paul_Jokiel/publication/270580749_Coral_growth_buoyant_weight_technique/links/54aec51d0cf29661a3d3ac96.pdf).

60. C. Ferrier-Pagès, C. Rottier, E. Beraud, O. Levy, Experimental assessment of the feeding effort of three scleractinian coral species during a thermal stress: Effect on the rates of photosynthesis. *J. Exp. Mar. Bio. Ecol.* **390**, 118–124 (2010).

61. J. E. Palardy, A. G. Grottoli, K. A. Matthews, Effects of upwelling, depth, morphology and polyp size on feeding in three species of Panamanian corals. *Mar. Ecol. Prog. Ser.* **300**, 79–89 (2005).

62. J. E. Palardy, A. G. Grottoli, K. A. Matthews, Effect of naturally changing zooplankton concentrations on feeding rates of two coral species in the Eastern Pacific. *J. Exp. Mar. Bio. Ecol.* **331**, 99–107 (2006).

63. P. J. Edmunds, R. Gates, Normalizing physiological data for scleractinian corals. *Coral reefs*. **21**, 193–197 (2002).

64. R-Core-Team, R: A language and environment for statistical computing. (2020), (available at https://www.r-project.org/).

65. M. J. Anderson, R. N. Gorley, K. R. Clarke, PRIMER v6: User Manual/Tutorial (Plymouth Routines in Multivariate Ecological Research). PRIMER-E, Plymouth (2006).

# Supplemental Tables

**Table S1** Summary of coral species, collection location, genets, and ramets collected as part of this study.

| Species | Site | Number of genets sampled per site | Number of ramets collected per genet | Number of coral fragments collected per site |
| --- | --- | --- | --- | --- |
| *Montipora capitata* | Moku o Loʻe | 6 | 4 | 24 |
|  | Waimānalo | 6 | 4 | 24 |
|  | Sampan | 6 | 4 | 24 |
|  | Haleʻiwa | 6 | 4 | 24 |
| *Porites compressa* | Moku o Loʻe | 6 | 4 | 24 |
|  | Waimānalo | 6 | 4 | 24 |
|  | Sampan | 6 | 4 | 24 |
|  | Haleʻiwa | 6 | 4 | 24 |
| *Porites lobata* | Waimānalo | 6 | 4 | 24 |
|  | Sampan | 6 | 4 | 24 |
|  | Haleʻiwa | 6 | 4 | 24 |
| Total number of coral fragments collected | | | | 264 |

**Table S2.** Meta-data of experimental methods. Reporting methods adapted from Grottoli et al. (2021). All dates in DD-MM-YYYY format.

| CORAL COLLECTION | **Latitude and longitude of collection sites:** | **Collection depth:** | **Collection dates:** | **Coral species and morphology:** | **Symbiodiniaceae for all coral species:** | **Acclimation prior to experiment:** |
| --- | --- | --- | --- | --- | --- | --- |
|  | Moku o Loʻe:  21.434167 N,  -157.786335 W  Waimānalo:  21.326287 N,  -157.674599 W  Sampan:  21.452394 N,  -157.794870 W  Haleʻiwa:  21.592516 N,  -158.110337 W | 2 ± 1 m | Between 29-08-2015 and 11-11-2015 | *Montipora* *capitata* (branching and encrusting)*,*  *Porites compressa* (branching)*,*  *Porites lobata* (massive) | *Cladocopium* in *Porites compressa* and *Porites lobata. Durusdinium* and *Cladocopium* in *Montipora capitata*. | At least 82 days, from 11-11-2015 to 01-02-2016 |
| EXPERIMENTAL DESIGN | **Name of experimental location:** | **Stress treatment period:** | **Tank system type:** | **Number of tanks per treatment:** | **Number of coral genets per treatment:** | **Number of recovery days post-stress:** |
|  | Hawaiʻi Institute of Marine Biology, Kāneʻohe, Hawaiʻi, USA | 20-02-2016 to 13-12-2017 | Outdoor, reef-derived, flow-through | 10 tanks | 18 to 24 genets | n/a |
| EXPERIMENTAL TEMPERATURE AND *p*CO_2_ CONDITIONS | **Stress temperature and *p*CO_2_ above MMM:** | **Control temperature and *p*CO_2_:** | **Baseline temperature and *p*CO_2_:** | **Temperature and *p*CO_2_ ramp-up rate:** | **Duration at stress**  **Temperature and/or *p*CO_2_ stress level:** | **Temperature and *p*CO_2_ modulation:** |
|  | +2 ℃ and/or + 350 µatm depending on treatment | ~23.5–27.5 ˚C over the annual cycle and ~400 μatm *p*CO_2_ | ~23.5–27.5 ˚C over the annual cycle and ~400 μatm *p*CO_2_ | 0.5 °C and -0.05 pH unit increments every 10 days | 662 days | Diurnally and seasonally varying |
| OTHER EXPERIMENTAL CONDITIONS | **Light conditions & cycle:** | **Flow rate**  **(cm s^-1^):** | **Tank turnover rate:** | **Seawater source and filtration:** | **Other abiotic variables** | **Coral feeding:** |
|  | Outdoor with 30% shade cloth. Diurnally and seasonally varying | Not measured | Inflow rate was ~1.2 L min^-1^ with a residence time of 1 hour | Natural seawater pumped directly from adjacent reef, unfiltered | Salinity: ~34.47–34.68 PSU  Nutrients: ~0.45–0.65 µmol PO_4_^3-^ and ~0.3–1.3 µmol NO_3_^-^  Dissolved Oxygen: ~6.6–7.5 mg O_2_ L^-1^ | No supplemental coral feeding, fish fed frozen mysid and adult brine shrimp daily |

**Table S3.** Comparison of coral future ocean dual stress experiments (i.e., combined ocean warming and acidification) which are > 30 days in duration that are published to date. Rows are ordered based on the duration of experimental stress (longest to shortest). SW = seawater, Ref = reference.

| **Ref.** | **Number of species, life-stage studied, and name of Scleractinian coral species** | **Number and name of coral collection location(s)** | **Experimental location** | **Mesocosm design (yes or no)** | **Temperature elevation from baseline** | ***p*CO_2_ elevation from baseline** | **Duration of treatment exposure (days)** | **Tank system type** | **Measured coral response variables** |
| --- | --- | --- | --- | --- | --- | --- | --- | --- | --- |
| This study | 3 (adult)  *Montipora capitata*  *Porites compressa*  *Porites lobata* | 4 reefs  Moku o Loʻe, Oʻahu, Hawaiʻi, USA  Sampan, Oʻahu, Hawaiʻi, USA  Waimānalo, Oʻahu, Hawaiʻi, USA  Haleʻiwa, Oʻahu, Hawaiʻi, USA | Oʻahu, Hawaiʻi, USA | yes | +2 ℃ | +350 µatm | 662 days | 40 outdoor tanks flow-through SW natural SW | coral mortality  coral color  coral endosymbiont density  coral photosynthesis  coral respiration  coral calcification  coral lipids  coral protein  coral tissue biomass  coral feeding capacity  coral total organic carbon flux  coral carbon budget |
| Bahr et al. (2020)^1^ | 1 (juvenile)  *Pocillopora acuta* | NA (*P. acuta* naturally recruited into the tanks) | Oʻahu, Hawaiʻi, USA | yes | +2 ℃ | +350 µatm | 662 days | 40 outdoor tanks flow-through SW natural SW | recruit abundance  recruit size  recruit mortality |
| Dove et al. (2020)^2^ | 8 (adult)  *Acropora formosa Seriatopora hystrix Stylophora pistillata Porites cylindrica Montipora sp.*  *Goniastrea aspera Lobophyllia sp.*  *Fungi asp.* | 1 reef  Heron Island reef flat, Great Barrier Reef, Australia | Heron Island, Great Barrier Reef, Australia | yes | +3.5 ℃ | +550 µatm | ~540 days  18 months | 12 outdoor tanks flow-through SW  natural SW | net ecosystem calcification  net ecosystem photosynthesis  net ecosystem respiration  benthic community composition |
| Dove et al (2013)^3^ | 8 (adult)  *Acropora formosa Seriatopora hystrix Stylophora pistillata Porites cylindrica Montipora sp.*  *Goniastrea aspera Lobophyllia sp.*  *Fungi asp.* | 1 reef  Heron Island reef flat, Great Barrier Reef, Australia | Heron Island, Great Barrier Reef, Australia | yes | - 1 ℃  +2 ℃  +4 ℃ | -104 µatm  +174 µatm +572 µatm | 85 days | 12 outdoor tanks flow-through SW  natural SW | net ecosystem calcification  coral calcification  net ecosystem oxygen flux  benthic community composition  sediment grain size  sediment-associated microbial community |
| Horvath et al. (2016)^4^ | 1 (adult)  *Siderastrea siderea* | 1 reef  Sapodilla Cayes Marine Reserve, Meso-American Barrier Reef System, Belize | North Carolina, USA | no | +4 ℃ | +516 ppm (approx.) | 60 days | 12 indoor tanks  recirculating SW  artificial SW | coral color (colorimetric reference card)  coral calcification  corallite morphology |
| Bahr et al. (2016)^5^ | 5 (adult)  *Porites compressa Pocillopora damicornis*  *Fungia scutaria*  *Montipora capitata*  *Leptastrea purpurea* | 1 reef  Moku o Loʻe, Oʻahu, Hawaiʻi USA | Oʻahu, Hawaiʻi, USA | yes | +2 ℃ | +520 µatm (approx.)  Specifically2 x ambient | 56 days | 4 outdoor tanks  flow-through SW natural SW | coral calcification |
| Brown et al. (2019)^6^ | 1 (adult)  *Acropora intermedia* | 1 reef  Heron Island reef flat, Great Barrier Reef, Australia | Heron Island, Great Barrier Reef, Australia | no | +3.5 ℃ | +572 µatm | 55 days in winter experiment  39 days in summer experiment | 24 outdoor tanks  flow-through SW natural SW | coral mortality  coral calcification  coral endosymbiont density  coral protein  coral photosynthesis  coral respiration |
| Van der Zande et al (2019)^7^ | 2 (adult)  *Acropora intermedia*  *Porites lobata* | 1 reef  Heron Island reef flat, Great Barrier Reef, Australia | Heron Island, Great Barrier Reef, Australia | no | +3.5 ℃ | +570 µatm | 49 days/  7 weeks | 16 outdoor tanks  flow-through SW  natural SW | coral mortality  coral calcification  coral endosymbiont density  coral protein  coral lipid  coral photosynthesis  coral respiration |
| Foster et al. (2015)^8^ | 1 (juvenile)  *Acropora spicifera* | 1 reef  Basile Island in the Southern Group of the Abrolhos Islands, Western Australia | Western Australia | no | +3 ℃ | +650 µatm | 35 days/  5 weeks | 4 indoor tanks, flow-through SW natural SW | larval settlement rate  larval post-settlement mortality  recruit calcification (skeletal weight) |
| ^1^ Bahr KD, Tran T, Jury CP, Toonen RJ (2020) Abundance, size, and survival of recruits of the reef coral Pocillopora acuta under ocean warming and acidification. PLoS One 15:1–13  ^2^ Dove SG, Brown KT, Van Den Heuvel A, Chai A, Hoegh-Guldberg O (2020) Ocean warming and acidification uncouple calcification from calcifier biomass which accelerates coral reef decline. Commun Earth Environ 1:1–9  ^3^ Dove SG, Kline DI, Pantos O, Angly FE, Tyson GW, Hoegh-Guldberg, O (2013) Future reef decalcification under a business-as-usual CO_2_ emission scenario. PNAS, 110, 15342–15347  ^4^ Horvath KM, Castillo KD, Armstrong P, Westfield IT, Courtney T, Ries JB (2016) Sci Reports, 6, 1–12  ^5^ Bahr KD, Jokiel PL, Rodgers KS (2016) Relative sensitivity of five Hawaiian coral species to high temperature under high-*p*CO_2_ conditions. Coral Reefs 35:729–738  ^6^ Brown KT, Bender-Champ D, Kenyon TM, Remond C, Hoegh-Guldberg O, Dove S (2019) Temporal effects of ocean warming and acidification on coral algal competition. Coral Reefs, 38, 297–309  ^7^ Van der Zande R, Achlatis M, Bender-Champ D, Kubicek A, Dove SG, Hoegh-Guldberg O (2019) Paradise lost: End-of-century warming and acidification under business-as-usual emissions have severe consequences for symbiotic corals. Global Change Biology, 26, 2203–2219  ^8^ Foster T, Gilmour JP, Chua CM, Falter JL, McCulloch MT (2015) Effect of ocean warming and acidification on the early life stages of subtropical Acropora spicifera. Coral Reefs, 34, 1217–1226 | | | | | | | | | |

**Table S4.** Equations used in the calculations of a) calcification, b) photosynthesis and respiration, c) total organic carbon flux, d) carbon budget parameters, and e) maximum *Artemia* capture rate. **Equations are in bold**. *Notes are in italics.*

|  | Value | Description | Units | **Equations** and *Notes* |
| --- | --- | --- | --- | --- |
| a | AW_T1_ | Air weight of coral at start of experiment | mg | *See: Jokiel et al. 1978.* |
|  | AW_T2_ | Air weight of coral at end of experiment | mg |  |
|  | G | Calcification | mg CaCO_3_/g/day | **[AW_T1_ - AW_T2_] / [AW_T1_ * # days between T1 and T2]** |
| b | ΔO_2-coral_ | Change in oxygen concentration in coral chamber during incubation | g O_2_/L/min | *The slope of the regression line between dissolved oxygen values measured in the incubation chamber (g/L) and time (min).* |
|  | ΔO_2-blank_ | Change in oxygen concentration in blank chamber during incubation | g O_2_/L/min | *The slope of the regression line between dissolved oxygen values measured in the incubation chamber (g/L) and time (min).* |
|  | vol | volume of seawater in the incubation chamber | L | *NA* |
|  | AFDW | Ash-free dry weight of the coral sample | gdw | *NA* |
|  | P_net_ | Net photosynthesis | µmol O_2_/hr/gdw | **[[[**Δ**O_2-coral_*vol] – [**Δ**O_2-blank_*vol]] / AFDW]*60min/31.9988*10^6^**  *Dividing by 31.9988 converts grams into moles of oxygen.* |
|  | P_gross_ | Gross photosynthesis | µmol O_2_/hr/gdw | **P_net_ + [ABS[R_d_ ]]** |
|  | P_net-C_ | Net photosynthesis in carbon units | g C/day/gdw | **[[[**Δ**O_2-coral_*vol] – [**Δ**O_2-blank_*vol]] / AFDW]*60min*11hr*0.375**  *Multiplying by 0.375 converts grams of oxygen into grams of carbon. Multiplying by 11hrs corrects for the number of daylight hours.* |
|  | P_gross-C_ | Gross photosynthesis in carbon units | g C/day/gdw | **P_net-C_ + [ABS[R_d-C_ ]]** |
|  | R_d_ | Day respiration | µmol O_2_/hr/gdw | **ABS[[[[**Δ**O_2-coral_*vol] – [**Δ**O_2-blank_*vol]] / AFDW]*60min/31.9988*10^6^]**  *Dividing by 31.9988 converts grams into moles of oxygen.* |
|  | R_d-C_ | Day respiration in carbon units | g C/day/gdw | **ABS[[[**Δ**O_2-coral_*vol] – [**Δ**O_2-blank_*vol]] / AFDW]*60min*11hr*0.375**  *Multiplying by 0.375 converts grams of oxygen into grams of carbon. Multiplying by 11hrs corrects for the number of daylight hours.* |
|  | R_n_ | Night respiration | µmol O_2_/hr/gdw | **ABS[[[[**Δ**O_2-coral_*vol] – [**Δ**O_2-blank_*vol]] / AFDW]*60min/31.9988*10^6^]**  *Dividing by 31.9988 converts grams into moles of oxygen.* |
|  | R_n-C_ | Night respiration in carbon units | g C/day/gdw | **ABS[[[**Δ**O_2-coral_*vol] – [**Δ**O_2-blank_*vol]] / AFDW]*60min*13hr*0.375**  *Multiplying by 0.375 converts grams of oxygen into grams of carbon. Multiplying by 13hrs corrects for the number of nighttime hours.* |
|  | R_t_ | Total respiration | µmol O_2_/hr/gdw | **[[R_d_*11hr] + [R_n_*13hr]]/24hr**  *In Dec 2018 there were 11 hours of daylight and 13 hours of night.* |
|  | R_t-C_ | Total respiration in carbon units | g C/day/gdw | **[R_d-C_] + [R_n-C_]** |
| c | TOC_-coral_ | Concentration of total organic carbon in coral chamber at the end of incubation | g C/L | *NA* |
|  | TOC_-blank_ | Concentration of total organic carbon in blank chamber at the end of incubation | g C/L | *NA* |
|  | dur | Duration of incubation | min | *NA* |
|  | TOC Flux | Total organic carbon release or uptake by coral in molar units | µmol C/hr/gdw | **[[[TOC_-coral_*vol] – [TOC_-blank_*vol]] / dur] / AFDW]*60min/12.0107*10^6^**  *Dividing by 12.0107 converts grams into moles of carbon.* |
|  | TOC Flux-C | Total organic carbon release or uptake by coral in gram units | g C/hr/gdw | **[[[TOC_-coral_*vol] – [TOC_-blank_*vol]] / dur] / AFDW]*60min** |
| d | CZAR | Contribution of Zooxanthellae to Animal Respiration | % | **[P_gross-C_ / R_t-C_] * 100** |
|  | CHAR_TOC_ | Contribution of TOC Heterotrophy to Animal Respiration | % | **[TOC Flux-C * 13hr] / R_t-C_ *100** |
|  | CTAR | Contribution of Total Acquired carbon to daily Respiration | % | **CZAR + CHAR_TOC_** |
| e | Art_T0_ | The average number of *Artemia* in 10ml pipette (n=5) at time zero (i.e., start of incubation) | #*Artemia* in 10ml | **[# in pipet1 + # in pipet2 + # in pipet3 + # in pipet4 + # in pipet5] / 5** |
|  | Art_T40_ | The average number of *Artemia* in 10ml pipette (n=5) at end of incubation | #*Artemia* in 10ml | **[# in pipet1 + # in pipet2 + # in pipet3 + # in pipet4 + # in pipet5] / 5** |
|  | Dur | Duration of incubation | min | *NA* |
|  | ΔArt_-coral_ | Change in *Artemia* concentration in coral chamber during incubation | #*Artemia*/min | **[[Art_T0_ - Art_T40_]/10ml*vol(ml)]/dur** |
|  | ΔArt_-blank_ | Change in *Artemia* concentration in blank chamber during incubation | #*Artemia*/min | **[[Art_T0_ - Art_T40_]/10ml*vol(ml)]/dur** |
|  | Feeding | Maximum Artemia Capture Rate | #*Artemia*/min/gdw | **[**Δ**Art_-coral_ -** Δ**Art_-blank_] / AFDW** |

**Table S5.** Analysis of deviance tables for the generalized linear model to determine the effects of temperature, *p*CO_2_, and species on survivorship. An asterisk denotes the interaction between factors. Bolded p-values are significant.

|  | df | Deviance | Residual df | Residual deviance | Pr (>Chi) |
| --- | --- | --- | --- | --- | --- |
| **All corals** |  |  |  |  |  |
| Null |  |  | 263 | 287.8 |  |
| Temperature | 1 | 39.963 | 262 | 247.8 | **<0.0001** |
| *p*CO_2_ | 1 | 1.574 | 261 | 246.3 | 0.2096 |
| Species | 2 | 9.658 | 259 | 236.6 | **0.0080** |
| Temperature**p*CO_2_ | 1 | 0.694 | 258 | 235.9 | 0.4049 |
| Temperature*Species | 2 | 2.638 | 256 | 233.3 | 0.2674 |
| *p*CO_2_*Species | 2 | 1.629 | 254 | 231.6 | 0.4429 |
| Temperature**p*CO_2_*Species | 2 | 0.419 | 252 | 231.2 | 0.8109 |

**Table S6.** PERMANOVA test results for temperature and *p*CO_2_ fixed effects and header and mesocosm tank random effects on coral multivariate phenotypic traits for a) *Montipora capitata,* b) *Porites compressa,* and c) *Porites lobata*. P-values in bold are significant at alpha=0.05. Df=degrees of freedom, SS=sum of squares, MS=mean sum of squares. Permutation method: Permutation of residuals under a reduced model. Number of permutations: 9999.

| Species | | Df | Ss | Ms | Pseudo-f | P |
| --- | --- | --- | --- | --- | --- | --- |
| **a) *Montipora capitata*** | | | | | | |
|  | Temperature | 1 | 46.131 | 46.131 | 3.943 | **0.0096** |
|  | *p*CO_2_ | 1 | 10.342 | 10.342 | 0.884 | 0.5376 |
|  | Temperature**p*CO_2_ | 1 | 20.287 | 20.387 | 1.743 | 0.1442 |
|  | Header(temperature**p*CO_2_) | 4 | 48.469 | 12.117 | 1.289 | 0.1901 |
|  | Tank(header(temperature**p*CO_2_) | 27 | 255.740 | 9.472 | 1.150 | 0.2187 |
|  | Residuals | 29 | 238.870 | 8.237 |  |  |
|  | Total | 63 | 630.000 |  |  |  |
|  |  |  |  |  |  |  |
| **b) *Porites compressa*** | | | | | | |
|  | Temperature | 1 | 20.496 | 20.496 | 2.723 | **0.0159** |
|  | *p*CO_2_ | 1 | 33.735 | 33.735 | 4.482 | **0.0006** |
|  | Temperature**p*CO_2_ | 1 | 21.262 | 21.262 | 2.825 | **0.011** |
|  | Header(temperature**p*CO_2_) | 4 | 29.950 | 7.488 | 0.838 | 0.735 |
|  | Tank(header(temperature**p*CO_2_) | 29 | 255.300 | 8.804 | 0.886 | 0.834 |
|  | Residuals | 43 | 427.150 | 9.934 |  |  |
|  | Total | 79 | 790.000 |  |  |  |
|  |  |  |  |  |  |  |
| **c) *Porites lobata*** | | | | | | |
|  | Temperature | 1 | 16.178 | 16.178 | 2.717 | **0.0351** |
|  | *p*CO_2_ | 1 | 13.363 | 13.363 | 2.245 | 0.0630 |
|  | Temperature**p*CO_2_ | 1 | 32.020 | 32.020 | 5.379 | **0.0031** |
|  | Header(temperature**p*CO_2_) | 4 | 22.872 | 5.718 | 0.573 | 0.9552 |
|  | Tank(header(temperature**p*CO_2_) | 34 | 239.910 | 9.996 | 1.016 | 0.4634 |
|  | Residuals | 35 | 246.050 | 9.842 |  |  |
|  | Total | 56 | 560.000 |  |  |  |
|  |  |  |  |  |  |  |

**Table S7.** Pairwise PERMANOVA test results for treatment fixed effects on coral multivariate phenotypic traits for a) *Montipora capitata,* b) *Porites compressa,* and c) *Porites lobata*. P-values in bold are significant at alpha=0.05 with Benjamini-Hochberg correction for multiple comparisons. Permutation method: Unrestricted permutation of raw data. Number of permutations: 9999. When pairwise PERMANOVA test between the control and a stress treatment was significant, similarities percentage analysis (SIMPER) was used to identify which phenotypic traits explained the largest proportion of the variance. Df=degrees of freedom, SS=sum of squares, MS=mean sum of squares, CO=control, OA=ocean acidification, OW=ocean warming, FO=future ocean, Max=maximum.

| Species | | PERMANOVA | | | SIMPER |
| --- | --- | --- | --- | --- | --- |
|  |  | T | P | P_corrected_ |  |
| **a) *Montipora capitata*** | | | | | |
|  | CO vs. OA | 1.3697 | 0.0851 | 0.1021 |  |
|  | CO vs. OW | 1.5290 | 0.0413 | 0.0620 |  |
|  | CO vs. FO | 1.9716 | 0.0020 | **0.0060** | 19.98% Percent whiteness  14.91% TOC flux  12.07% Max *Artemia* capture rate |
|  | OA vs. OW | 1.8987 | 0.0038 | **0.0076** |  |
|  | OA vs. FO | 2.3944 | 0.0001 | **0.0006** |  |
|  | OW vs FO | 1.2496 | 0.1557 | 0.1557 |  |
|  |  |  |  |  |  |
| **b) *Porites compressa*** | | | | | |
|  | CO vs. OA | 2.3043 | 0.0001 | **0.0003** | 14.49% Symbiodiniaceae density  13.91% Protein  12.18% Total respiration |
|  | CO vs. OW | 2.0463 | 0.0001 | **0.0003** | 13.89% Max *Artemia* capture rate  12.76% Total organic carbon flux  12.13% Biomass |
|  | CO vs. FO | 1.9142 | 0.0009 | **0.0018** | 14.37% Gross photosynthesis  13.25% Total respiration  12.75% Symbiodiniaceae density |
|  | OA vs. OW | 1.6280 | 0.0095 | **0.0143** |  |
|  | OA vs. FO | 0.9716 | 0.4681 | 0.4681 |  |
|  | OW vs FO | 1.1600 | 0.2105 | 0.2526 |  |
|  |  |  |  |  |  |
| **c) *Porites lobata*** | | | | | |
|  | CO vs. OA | 1.5518 | 0.0254 | 0.0762 |  |
|  | CO vs. OW | 1.6072 | 0.0089 | 0.0534 |  |
|  | CO vs. FO | 1.2919 | 0.1326 | 0.1591 |  |
|  | OA vs. OW | 1.0087 | 0.4072 | 0.4072 |  |
|  | OA vs. FO | 1.4242 | 0.0620 | 0.1182 |  |
|  | OW vs FO | 1.3635 | 0.0788 | 0.1182 |  |

**Table S8.** One-way ANOVA results for treatment fixed effects for each coral phenotypic trait in *Montipora capitata* (MC)*, Porites compressa* (PC)*,* and *Porites lobata* (PL). When ANOVA assumptions of normality and heteroscedasticity could not be met, Kruskal Wallis one-way analysis of variance was used. P-values in bold are significant at alpha = 0.05. Trans = transformation, Df = degrees of freedom, SS = sum of squares.

|  | Species | ANOVA or Kruskal Wallis | | | | | |
| --- | --- | --- | --- | --- | --- | --- | --- |
|  |  | Source | Trans. | Df | SS | F or χ^2^ | P |
| Percent whiteness | | | | | | | |
|  | *MC* | Treatment |  | 3 | Kruskal | 16.528 | **0.0009** |
|  |  | residuals |  |  |  |  |  |
|  | *PC* | Treatment |  | 3 | 653 | 3.208 | **0.0277** |
|  |  | residuals |  | 76 | 5158 |  |  |
|  | *PL* | Treatment |  | 3 | Kruskal | 3.5358 | 0.3161 |
|  |  | residuals |  |  |  |  |  |
| Symbiodiniaceae density | | | | | | | |
|  | *MC* | Treatment | Log_10_(X) | 3 | 3.801 x10^15^ | 0.351 | 0.7880 |
|  |  | residuals |  | 60 | 2.165 x10^17^ |  |  |
|  | *PC* | Treatment |  | 3 | 5.195 x10^16^ | 3.215 | **0.0275** |
|  |  | residuals |  | 76 | 4.093 x10^17^ |  |  |
|  | *PL* | Treatment |  | 3 | 5.198 x10^16^ | 1.931 | 0.1360 |
|  |  | residuals |  | 53 | 4.756 x10^17^ |  |  |
| Gross photosynthesis | | | | | | | |
|  | *MC* | Treatment |  | 3 | 5721 | 2.683 | 0.0547 |
|  |  | residuals |  | 60 | 42641 |  |  |
|  | *PC* | Treatment |  | 3 | Kruskal | 12.685 | **0.0054** |
|  |  | residuals |  | 76 |  |  |  |
|  | *PL* | Treatment | Log_10_(X) | 3 | 1.172 | 1.905 | 0.1400 |
|  |  | residuals |  | 53 | 10.871 |  |  |
| Calcification | | | | | | | |
|  | *MC* | Treatment | Log_10_(X) | 3 | 0.810 | 0.395 | 0.7570 |
|  |  | residuals |  | 60 | 40.81 |  |  |
|  | *PC* | Treatment | Log_10_(X) | 3 | 2.459 | 1.996 | 0.1220 |
|  |  | residuals |  | 76 | 31.211 |  |  |
|  | *PL* | Treatment |  | 3 | 193.9 | 2.649 | 0.0583 |
|  |  | residuals |  | 53 | 1293.4 |  |  |
| Total respiration | | | | | | | |
|  | *MC* | Treatment | Log_10_(X) | 3 | 1.688 | 5.887 | **0.0014** |
|  |  | residuals |  | 60 | 5.736 |  |  |
|  | *PC* | Treatment |  | 3 | 119.8 | 3.457 | **0.0205** |
|  |  | residuals |  | 76 | 877.8 |  |  |
|  | *PL* | Treatment | Log_10_(X) | 3 | 1.272 | 1.838 | 0.1520 |
|  |  | residuals |  | 53 | 12.231 |  |  |
| Total organic carbon flux | | | | | | | |
|  | *MC* | Treatment | Log_10_(X + 40) | 3 | 18.39 | 14.21 | **<0.0001** |
|  |  | residuals |  | 60 | 25.89 |  |  |
|  | *PC* | Treatment |  | 3 | Kruskal | 8.8086 | **0.0319** |
|  |  | residuals |  |  |  |  |  |
|  | *PL* | Treatment | Log_10_(X + 150) | 3 | 3.006 | 1.848 | 0.1500 |
|  |  | residuals |  | 53 | 28.735 |  |  |
| Biomass | | | | | | | |
|  | *MC* | Treatment | Log_10_(X) | 3 | 1.878 | 4.029 | **0.0112** |
|  |  | residuals |  | 60 | 9.322 |  |  |
|  | *PC* | Treatment |  | 3 | 190 | 3.251 | **0.0263** |
|  |  | residuals |  | 76 | 1480 |  |  |
|  | *PL* | Treatment |  | 3 | Kruskal | 8.1571 | **0.0429** |
|  |  | residuals |  |  |  |  |  |
| Lipid | | | | | | | |
|  | *MC* | Treatment |  | 3 | Kruskal | 3.0882 | 0.3782 |
|  |  | residuals |  |  |  |  |  |
|  | *PC* | Treatment |  | 3 | Kruskal | 9.1917 | **0.0269** |
|  |  | residuals |  |  |  |  |  |
|  | *PL* | Treatment |  | 3 | Kruskal | 7.8682 | **0.0488** |
|  |  | residuals |  |  |  |  |  |
| Protein | | | | | | | |
|  | *MC* | Treatment | Log_10_(X) | 3 | 0.316 | 1.513 | 0.2200 |
|  |  | residuals |  | 60 | 4.183 |  |  |
|  | *PC* | Treatment | Log_10_(X) | 3 | 0.499 | 0.759 | 0.5210 |
|  |  | residuals |  | 76 | 16.678 |  |  |
|  | *PL* | Treatment |  | 3 | 547 | 0.247 | 0.8630 |
|  |  | residuals |  | 53 | 39159 |  |  |
| Maximum *Artemia* capture rate | | | | | | | |
|  | *MC* | Treatment | Log_10_(X) | 3 | 2.34 | 0.655 | 0.5830 |
|  |  | residuals |  | 60 | 71.58 |  |  |
|  | *PC* | Treatment |  | 3 | Kruskal | 9.1347 | **0.0276** |
|  |  | residuals |  |  |  |  |  |
|  | *PL* | Treatment | Log_10_(X) | 3 | 3.212 | 3.56 | **0.0202** |
|  |  | residuals |  | 53 | 15.941 |  |  |
| Contribution of Total acquired carbon to Animal Respiration (CTAR) | | | | | | | |
|  | *MC* | Treatment |  | 3 | Kruskal | 26.678 | **<0.0001** |
|  |  | residuals |  |  |  |  |  |
|  | *PC* | Treatment |  | 3 | Kruskal | 7.8172 | **0.0499** |
|  |  | residuals |  |  |  |  |  |
|  | *PL* | Treatment |  | 3 | Kruskal | 3.4656 | 0.3253 |
|  |  | residuals |  |  |  |  |  |

# Supplemental Figures


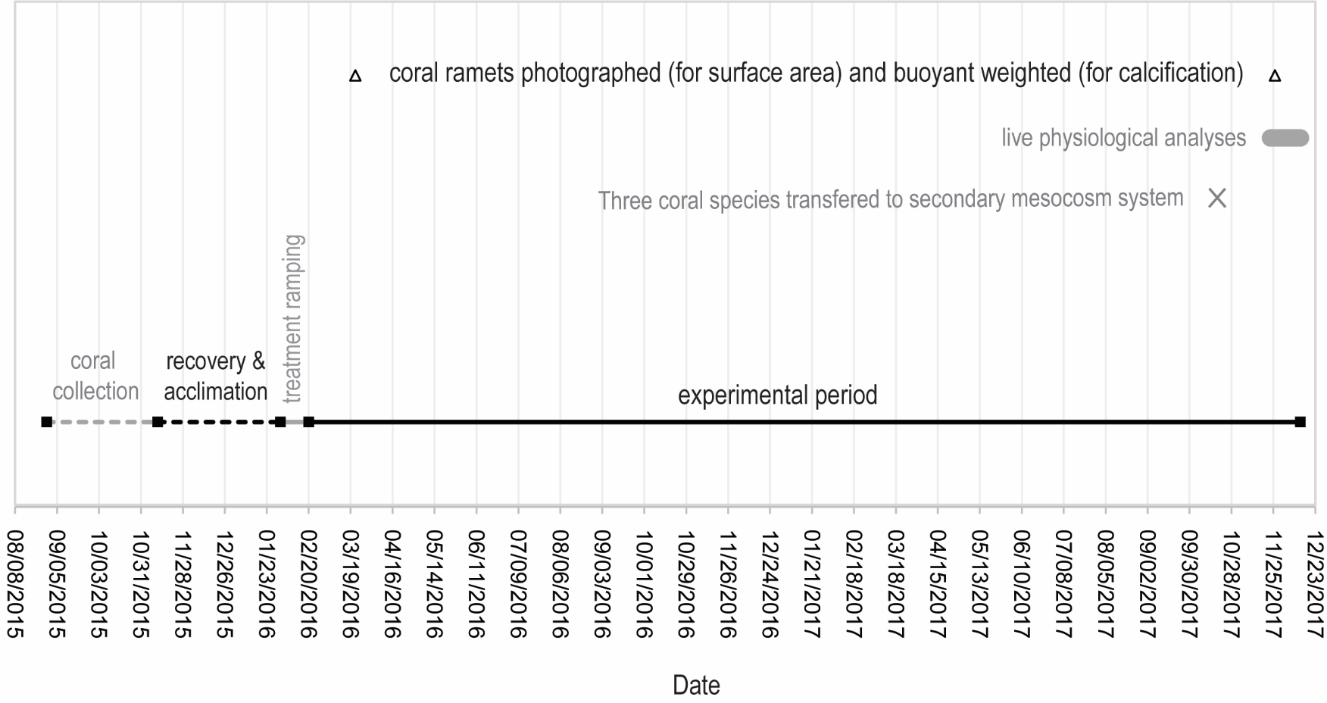


**Fig. S1.** Timeline of experimental procedures and live coral physiological measurements between August 2015 and December 2017. Dates provided as MM/DD/YYYY.
